# ENY2 transcription and export complex 2 subunit deficiency induces nucleolar stress to inhibit tumor progression through NPM1/MDM2/p53-dependent and -independent responses

**DOI:** 10.1007/s13402-025-01148-4

**Published:** 2026-02-05

**Authors:** Shiqi Zuo, Siyuan He, Zhiqin Zhu, Yanjie Hou, Ziqing Wu, Yao Tang, Yujiao Zou

**Affiliations:** 1 Department of Pathology, Southern Medical University Hospital of Integrated Traditional Chinese and Western Medicine, School of Basic Medical Sciences, Southern Medical University, Guangzhou, Guangdong China; 2 Guangdong Province Key Laboratory of Molecular Tumor Pathology, Guangzhou, Guangdong China; 3 Center of Respiratory Medicine, Xiangya Hospital Central South University, Changsha, Hunan China; 4 Department of Radiation oncology, Zhujiang Hospital, Southern Medical University, Guangzhou, Guangdong China

**Keywords:** ENY2 transcription and export complex 2 subunit, NPM1, Nucleolar stress, p53-independent response pathway, anti-IL11 therapy

## Abstract

**Purpose:**

The selective induction of nucleolar stress in cancer cells has become a potential anticancer therapy. However, precisely regulating the key molecules involved in nucleolar stress remains a challenging topic in current research. ENY2 transcription and export complex 2 subunit (ENY2) is a transcription-associated nuclear protein that is upregulated in several cancers. However, its specific function and mechanistic role in oncogenesis remain poorly characterized and require further exploration.

**Methods:**

ENY2 was identified by screening ChIP-seq and public databases. Its role in tumor development was confirmed through in vivo and in vitro experiments. RNA sequencing, polysome profiling, agarose gel electrophoresis, and immunofluorescence suggested ENY2’s involvement in ribosome biogenesis. Interacting proteins were identified by confocal microscopy, co-IP, and molecular docking, then validated by western blotting and ubiquitination assays. Finally, drug resistance experiments evaluated ENY2’s clinical potential.

**Results:**

We discovered that the overexpression of ENY2 significantly enhances tumor growth and cell cycle progression both in vitro and in vivo. Conversely, depletion of ENY2 facilitating the release of NPM1 into the nucleoplasm, thereby impeding ribosomal subunit export and inducing nucleolar stress. Additionally, the released NPM1 interacts with MDM2 within the nucleus to stabilize p53 protein levels, consequently inhibiting tumor growth. Notably, knockdown of ENY2 in p53-mutant cancer cell lines exhibits an augmented binding affinity and silencing efficacy of RISC towards target mRNA molecules, ultimately suppressing tumor proliferation through a p53-independent manner.

**Conclusions:**

This study elucidated a previously unrecognized role of ENY2 in tumor growth, clarified the NPM1/MDM2/ p53-dependent mechanism of ENY2-mediated tumor cell growth suppression. We also provided a novel p53-independent RISC-IL11 nucleolar stress response pathway, which may provide a new target for the treatment of breast cancer.

**Graphical Abstract:**

**Figure:**
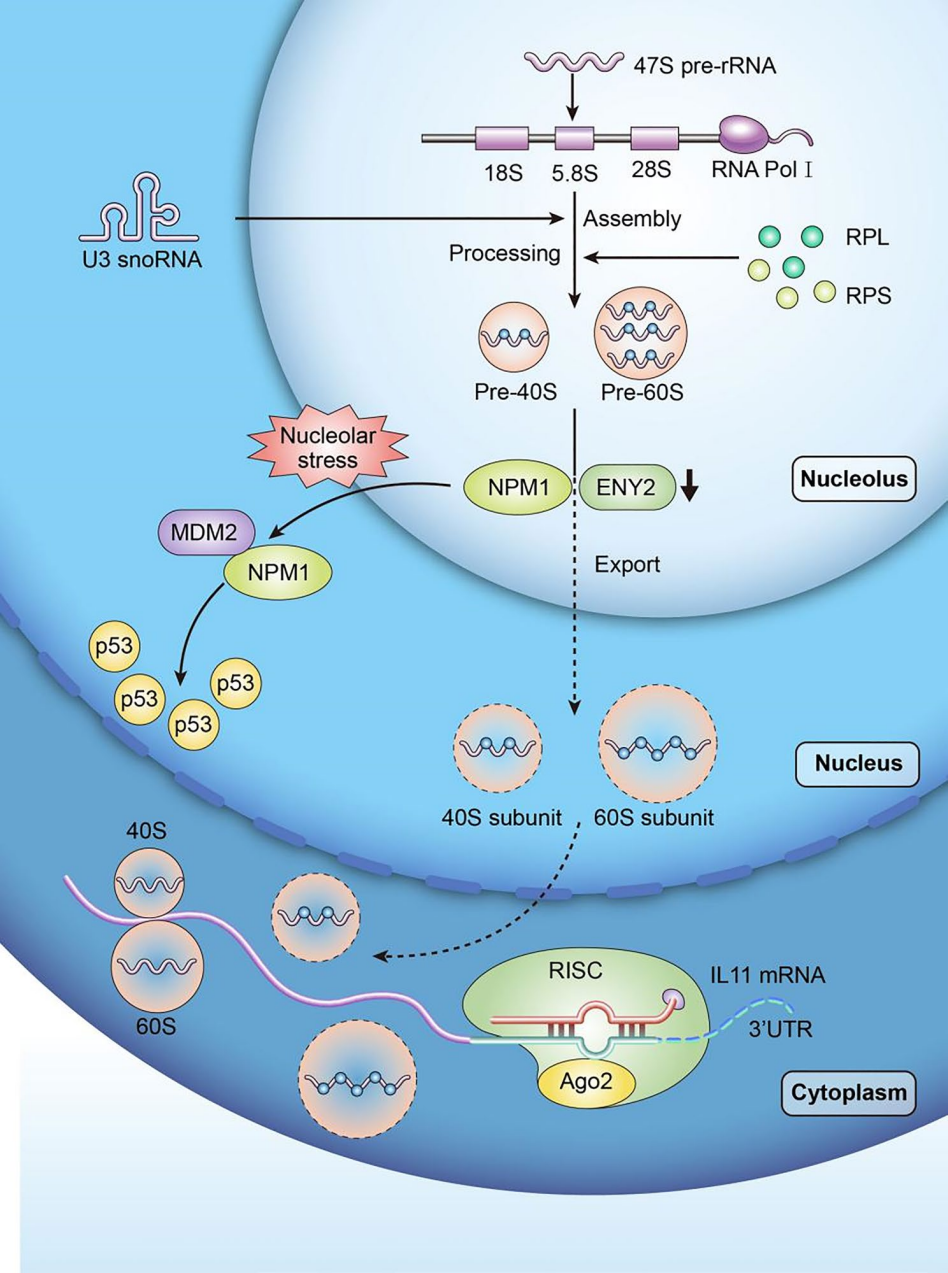
ENY2 promoting tumor initiation and progression by inducing p53-dependent and independent nucleolar stress responses

**Supplementary Information:**

The online version contains supplementary material available at 10.1007/s13402-025-01148-4.

## Introduction

The nucleolus is a large nuclear membraneless compartment principally engaged in the synthesis of ribosomal subunits and encompasses several distinct nucleolar domains [1]. This process is tightly controlled, involving the transcription of ribosomal DNA (rDNA) and the subsequent processing of precursor rRNA [2, 3]. Subsequently, the granular components (GC) are involved in the final stages of rRNA maturation and ribosomal subunit assembly [4]. Nucleolar stress is a cellular state triggered by disruptions in ribosome biogenesis, leading to significant alterations in nucleolar morphology and function. This stress arises from errors in key processes such as ribosomal RNA synthesis, processing, or ribosomal subunit assembly. Consequently, nucleolar stress activates downstream pathways that can induce cell cycle arrest or apoptosis, depending on the extent of damage and cellular context [5, 6]. The well-established nucleolar stress response mechanism is primarily orchestrated by the tumor suppressor protein p53. Under nucleolar stress, pivotal ribosomal proteins such as RPL5 and RPL11 are liberated from the nucleolus into the nucleoplasm, where they interact with MDM2. This interaction impedes MDM2’s capacity to bind and ubiquitinate p53, thereby enhancing p53’s stability and activity [7]. This activation cascade subsequently upregulates the expression of the cell cycle inhibitor p21 and pro-apoptotic genes, leading to cell cycle arrest and apoptosis [8].

Cancer cells often require overactivated ribosome biogenesis to maintain rapid growth and proliferation [9], and the selective induction of nucleolar stress in highly metabolic cancer cells has become a potential therapeutic approach for cancer [10]. However, over 50% of human cancers exhibit non-functional p53 [11]. For example, there are extensive p53 mutations and inactivation in triple-negative breast cancer (TNBC). Missense mutations not only eliminate the tumor suppressor function of p53, but also often enable the acquisition of new tumorigenic driving activity [12, 13]. Because of the high rate of p53 inactivation, therapeutic methods intended to target p53 and MDM2 have little effect on TNBC [14]. Of note, researchers have found that yeast does not express characteristic p53 or MDM2 [15, 16], similar to the lack of genes encoding MDM2 and ARF in Drosophila and other metanozoans [17]; however, the nucleolus stress response pathway is still observed in these ancient organisms [18–20]. This inquiry led us to explore the possibility of p53-independent response mechanisms in human cells and, if present, whether these could be harnessed to address the majority of human cancers that exhibit non-functional p53. Remarkably, past research has indicated that nucleolar stress can trigger p53-independent pathways, including the degradation of the RNA polymerase I (Pol I) complex, which subsequently activates the NF-κB pathway. This activation has been linked to the anti-tumor properties of aspirin [21]. Additionally, studies have demonstrated that Protein Arginine Methyltransferase 1 (PRMT1) induces arginine methylation of p14ARF, resulting in its relocalization to the nucleolus and exerting a p53-independent tumor suppressive effect [22]. A growing body of evidence suggests that the p53-independent nucleolar stress pathway represents a promising novel avenue for cancer therapy. Furthermore, free ribosomal proteins have been shown to modulate the growth, migration, and autophagy of cancer cells via nucleolar stress-induced signaling pathways [23]. Nonetheless, the cellular mechanisms underlying this stress response remain incompletely elucidated.

In the context of our research, we observed that ENY2 transcription and export complex 2 subunit (ENY2), plays a pivotal role in both p53-dependent and p53-independent pathways following nucleolar stress. ENY2 is a nuclear protein associated with transcription, which has been identified as a critical element within the Spt-Ada-GCN5-Acetyltransferase (SAGA) complexes [24, 25]. These complexes are integral to processes such as transcriptional activation and histone deubiquitination, thereby influencing gene expression patterns [26–28]. Accumulating evidence have demonstrated that ENY2 is significantly overexpressed at the transcriptional and protein levels in multiple cancer types relative to adjacent normal tissues [29–31]. The aberrant overexpression of ENY2 is associated with modifications in histone H2B mono-ubiquitination (H2Bub) levels [32], disruptions in the H2B/H2Bub1 equilibrium, and compromised DNA repair mechanisms. These alterations contribute to the initiation and advancement of tumors [33]. Despite these findings, the precise roles and mechanisms of ENY2 in cancer progression remain largely unexplored and warrant further investigation.

Here, we identified that ENY2 is highly expressed in multiple cancers and associated with poor prognosis. Knockdown of ENY2 impairs the binding to nucleophosmin (NPM1), resulting in export impairment of 40 S and 60 S ribosomal subunits, inducing nucleolar stress and feed-back accumulation of precursor rRNA. NPM1 migrates from the nucleolus to the nucleoplasm and binds to MDM2, attenuating ubiquitination and degradation of p53 and thereby inhibiting tumor growth. Nucleolar stress activates well-established p53-dependent pathways, exemplified by ribosomal proteins (e.g., RPL5, RPL11) translocating to the nucleoplasm to stabilize p53 via MDM2 binding [34–36]. Additionally, emerging evidence reveals p53-independent signaling mechanisms involving non-ribosomal nucleolar proteins [37]. Here, we identify a novel p53-independent mechanism linking ENY2 depletion to nucleolar stress, which promotes RISC-mediated IL-11 mRNA decay and suppresses STAT3-driven tumor proliferation. We therefore conclude that ENY2 depletion triggers a novel crosstalk linking nucleolar stress to ribosomal protein and IL-11 pathways, which in turn inhibits tumor growth through both p53-dependent and -independent mechanisms.

In summary, our study demonstrates ENY2 is a novel regulator of p53-dependent nucleolar stress and IL-11 signaling, and knockdown of ENY2 may suggest novel cancer treatment approaches targeting ribosome biogenesis.

## Materials and methods

### Cell culture

Cancerous and non-cancerous cell lines, including the human normal mammary epithelial cell line MCF-10 A; breast cancer cell lines T47D, SKBR3, MCF-7, HCC-1937, MDA-MB-231, and BT-549; colorectal cancer cell lines RKO and SW480; and human embryonic kidney cell line HEK-293T, were procured from Procell (Wuhan, China). These cell lines were propagated in Dulbecco’s Modified Eagle’s Medium (DMEM) (Gibco, Grand Island, NY, USA), which was enriched with 10% fetal bovine serum (Zeta life, USA). The cells were incubated at 37 °C within an environment containing 5% CO2. Additionally, all culture media were fortified with a 100x penicillin-streptomycin solution (Solarbio, Beijing, China) during routine cell cultivation.

### Data collection and bioinformatics analysis

The expression patterns of ENY2 transcription and export complex 2 subunit (ENY2) across 25 distinct cancer types were extracted from the TCGA and UALCAN repositories. The cBioPortal platform was employed to scrutinize the mutation spectrum of ENY2 across various cancer entities. Additionally, the CancerSEA platform elucidated the interplay between ENY2 and the biological characteristics of tumors, particularly in breast cancer, by leveraging the Metascape database to conduct a functional enrichment analysis on co-expressed genes within the context of breast cancer.

### siRNA/shRNA, lentivirus and plasmid transfection

Synthetic siRNAs against ENY2, NPM1, and siNC were purchased fromRiboBio (siBDM1999A, Guangzhou, China), and siRNA against Ago2 were designedand synthesized by OBio (RY24000251, Shanghai, China). Opti-MEM (Gibco, USA)and Lipofectamine 3000 transfection reagent (Invitrogen Biotechnology,Shanghai, China) were used to transfect the siRNA. For each gene, at least twodifferent siRNA sequences were tested, and the most effective one was selectedfor further experiments to ensure reproducibility and minimize off-targeteffects. Functional experiments were performed 48 h after transfection, andproteins were extracted 72 h after transfection. The ENY2 overexpressionplasmid pTSB was acquired from TranSheepBio (Shanghai, China), and thepSLenti-puro plasmids encoding shRNA targeting ENY2 were procured from Obio.The procedures for lentiviral packaging were performed as previously described [38].Cells were cultured in a mixture of complete medium supplemented withPolybrene, followed by transduction with the viral supernatant. Stabletransfected cell lines were subsequently selected using puromycin (Beyotime,Shanghai, China).

### RNA extraction and qPCR

Column purification of RNA was performed using FOREGENE’s total RNA extraction kit (Chengdu, China). Reverse transcription was performed using the Evo M-MLV RT Premix Kit (Accurate Biotechnology, Hunan, China). Genomic DNA was removed by incubating 2 µL of gDNA Clean Reaction Mix Ver.2 with total RNA and RNase-free water in a 16 µL reaction at 42 °C for 2 min. Then, 4 µL of 5×Evo M-MLV RT Reaction Mix Ver.2 was added to make a 20 µL final volume. The reaction was carried out at 37 °C for 15 min, followed by inactivation at 85 °C for 5 s. The resulting cDNA was used immediately for qPCR. qRT-PCR was performed using the SYBR Green Pro Taq HS Premix III (High Rox) kit from Elk Biotechnology (Hunan, China). The 20 µL reaction system consisted of 10 µL of 2×SYBR Green Pro Taq HS Premix III, 0.4 µL of each forward and reverse primer (10 µM), an appropriate amount of cDNA template, and RNase-free water up to the total volume. The amplification protocol began with an initial pre-denaturation at 95 °C for 30 s, followed by 40 cycles of denaturation at 95 °C for 5 s and annealing/extension at 60 °C for 30 s. Melting curve analysis was subsequently conducted at 95 °C for 15 s, 60 °C for 60 s, and 95 °C for 15 s. Data are presented as relative mRNA expression levels normalized to GAPDH and calculated using the 2^(-ΔΔCt) method, with the values in the siNC (negative control) group set as 1. The sequences of the primers are presented in Supplementary Table 1.

### Western blot and co-immunoprecipitation

Cells were harvested and lysed on ice for 30 min using RIPA lysis buffer (Beyotime, China) supplemented with protease and phosphatase inhibitors (prepared at a ratio of 1:1:100, inhibitor: RIPA buffer). The lysates were then centrifuged at 12,000 ×g for 15 min at 4 °C to remove insoluble debris. The supernatant was collected, and protein concentration was determined using a BCA assay kit (Beyotime, China) according to the manufacturer’s instructions. All samples were adjusted to an equal concentration with lysis buffer for subsequent experiments.

For Western blot, 30 µg of protein per lane was loaded and separated on SDS-polyacrylamide gels (10%, 12.5%, or 15%). Following electrophoresis, the proteins were transferred onto PVDF membranes (Millipore). The membranes were blocked with 5% non-fat milk for 1 h at room temperature and then incubated with the indicated primary antibodies (diluted in blocking buffer) overnight at 4 °C. The next day, after washing three times with TBST solution (Leagene, Beijing, China), the membranes were incubated with secondary antibodies for 1 h at room temperature. After further washing, the protein bands were visualized using an ECL luminescent kit (Epizyme, Shanghai, China) and captured with a chemiluminescence imaging system. Image Lab software (Bio-Rad) was used for image acquisition and analysis. 

For the co-immunoprecipitation (Co-IP) assay, samples were lysed using Co-ip lysate (Thermo Fisher Scientific). 3–4 µg of antibody was added and the mixture was incubated on a rotator. Then, Protein A/G beads (Thermo Fisher Scientific) were added, and the sample mixture was kept for 40 min and then washed five times with washing buffer. The immunoprecipitated complexes were eluted by boiling in SDS loading buffer (Epizyme) for subsequent Western blot analysis. All antibodies used are listed in Supplementary Table 2.

### Immunohistochemical technique (IHC)

The IHC procedure was performed as previously described with minor modifications [39]. Briefly, paraffin-embedded sections were deparaffinized in xylene and rehydrated through a graded ethanol series. Antigen retrieval was carried out using EDTA buffer (zsbio, Bejing, China) with microwave heating for 8 min. Endogenous peroxidase activity was quenched by incubation with 3% H₂O₂ (Leagene) for 10 min. Sections were then blocked with 10% normal goat serum (zsbio) for 30 min at room temperature and incubated with ENY2 antibody (same catalog with WB, dilution 1:100) at 4 °C overnight. After washing, the sections were incubated with HRP-conjugated secondary antibody at 37 °C for 30 min. DAB substrate (Boster, Wuhan, China) was applied for color development, and counterstaining was performed with hematoxylin. Finally, the sections were dehydrated, cleared in xylene, and mounted with neutral balsam (Solarbio).

### CCK-8 assay

Approximately 1000 cells were counted and seeded into individual wells of a 96-well microplate. Following cell attachment, 10 µL of CCK-8 reagent (Yeasen, Shanghai, China) was introduced into each well. After adding the CCK-8 reagent, the plate was incubated for 2 h at 37 °C and the optical density at 450 nm was subsequently measured using a microplate spectrophotometer at 2, 24, 48, 72, 96, and 120 h.

### Plate cloning assay

Approximately 0.5 × 10^3^ cells per well were counted. The culture medium was discarded and stained with crystal violet (Beyotime). The results were quantified using ImageJ software.

### Flow cytometry was used for cell cycle analysis

According to the manufacturer’s (KeyGEN, Jiangsu, China) instructions [40], 500 µL of working solution was used to incubate per sample; the staining working solution was prepared by mixing Rnase A and PI working solution at a ratio of 1:9 before use; cells were washed once with PBS, collected by centrifugation at 2000 rpm for 5 min, and adjusted to a concentration of 1 × 10⁶/mL, and 1 mL of single-cell suspension was taken for use; the prepared single-cell suspension was centrifuged and the supernatant was discarded, 500 µL of 70% cold ethanol was added to fix the cells for 2 h to overnight, and stored at 4 °C. Before staining, the fixative was washed away with PBS, and then the cell suspension was filtered once through a 200-mesh sieve, with centrifugation wash conditions at 1000 rpm for 3 min; 500 µL of pre-prepared PI/RNase A staining working solution was added to the cells, and incubated at room temperature in the dark for 60 min; finally, detection was performed using a flow cytometer, and the red fluorescence signal at an excitation wavelength of 488 nm was recorded.

### Orthotopic transplantation model in nude mice

Female BALB/c nude mice were purchased from the Animal Laboratory Center of Southern Medical University. The animal experimental protocol was approved by the Experimental Animal Ethics Committee of Southern Medical University, and the study followed international ethical guidelines for animal experiments. Twenty BALB/c nude mice were randomly divided into two groups (n = 10 per group) using a random number table in each experiment. All mice were housed under specific pathogen-free (SPF) conditions. In this study, 5 × 106 cells stably expressing shNC and shENY2 were suspended in 100 µL saline and injected to the subcutaneous fat pad of mouse mammary glands. The body weight and tumor size of the mice were gauged every 3 days for a total treatment period of 21 days and the tumor volume was calculated in the following way: tumor volume (mm3) = 0.52 × (length × width2). If the maximum tumor load did not exceed 1500 mm, the mice were euthanized to remove the tumor for measurement and analysis.

### RNA Sequencing

48 h after transfection with siNC and siENY2, cells were sent for detection with TRIzol (NCM Biotech, Suzhou, China), and RNA-seq services were provided by HaploX (Shenzhen, China). Total RNA was extracted and qualified using NanoDrop™ One/OneC for purity, Qubit® 3.0 for concentration, and Agilent 4200 TapeStation for RIN evaluation. Sequencing libraries were constructed using an oligo(dT) bead-based mRNA capture kit, including cDNA synthesis, end repair, A-tailing, and adapter ligation. Libraries were size-selected for ~ 200 bp fragments with AMPure XP beads, and quality was verified by Kapa qPCR and Agilent 4200 TapeStation. Paired-end sequencing (PE150) was performed on an Illumina platform.

For bioinformatics analysis, quality-controlled reads were aligned to the reference genome using HISAT2. Gene-level counts were obtained and used for differential expression analysis, which included normalization, p-value and fold-change calculation, and multiple testing correction. Functional enrichment analysis of GO and KEGG terms was performed using clusterProfiler with a hypergeometric test.

### Agarose gel assay

In breast cancer cells transfected with siENY2, RNA was extracted 48 h later for agarose gel detection, dissolved in 60 mL of 1× TAE solution (Solarbio) with 0.9 g agarose powder (Beyotime), After boiling in microwave oven for 5 min each time, take out and shake well. According to the principle of the three boiling steps, a 1.5% agarose gel was prepared and poured into the mold for solidification. Each well contained 5 µg RNA and was subjected to constant pressure at 120 V for 20 min. The gel was placed under UV light for the analysis of the bands.

### Polyribosome analysis

Density gradient solution was prepared by adding 1 mL each of 15% (w/v) and 50% (w/v) sucrose solution (Solarbio) into gradient centrifuge tubes, and mRNA samples extracted after transfection with siNC and siENY2 were added to the surface. Specifically, cellular lysates were clarified by centrifugation at 10,000 ×g for 10 min at 4 °C. A total of 500 µg of RNA from each sample was loaded onto a 15–50% sucrose gradient prepared in lysis buffer. Gradients were ultracentrifuged at 40,000 rpm for 2.5 h using a Beckman SW65 rotor in an Optima XE-90 ultracentrifuge. After ultracentrifugation, the sample bands were collected, and the absorbance at 254 nm was recorded using a spectrophotometer for quantitative analysis as the following literature [41].

### Immunofluorescence staining

Prior to the staining process, 2 × 10^4^ cells were cultured on fluorescent-coated dishes. The fixative solution was subsequently discarded and neutralized using 1 mL of a 2 mg/mL glycine solution (Leagene, China). To facilitate penetration and blocking, 1 mL of 0.5% TritonX-100 was introduced at ambient temperature and allowed to incubate for 10 min on a shaker. Following this, the cells were diluted with the primary antibody solution, which included anti-ENY2 and anti-NPM1 at dilutions of 1:100. Subsequently, the corresponding secondary antibody was applied, and the membrane was incubated at room temperature for 1 h. The final step involved sealing the slides with DAPI anti-fluorescence quench (Solarbio) to prevent fluorescence quenching according to the previous article [39].

### Molecular docking

The resolved structures of ENY2 and NPM1 were obtained from the RCSB PDB database (https://www.rcsb.org/) [42], and PyMOL software ( https://pymol.org/) was used for preparation, including dehydration and hydrogenation [43]. The ClusPro platform was used for rigid docking to adjust the initial conformation of the two proteins [44], and RosettaDock (http://rosettadock.graylab.jhu.edu) was used for flexible docking to screen the proteins according to the score module built in Rosetta [45]. IigPlot (https://www.ebi.ac.uk/thornton-srv/software/LigPlus/) software was used to analyze bond formation between the two proteins [46]. Finally, Rosetta’s interface analyzer module was used to analyze the docking free energy between ENY2 and NPM1. Finally, PyMOL software was used to visualize the protein conformation after docking.

### Determination of half-life

After transfection of siNC and siENY2 into breast cancer cells, 100 µg/mL CHX (MCE, USA) was added at 0 min, 30 min, 60 min and 90 min before protein collection, and the change of p53 protein level was detected using Western blotting.

### Measurement of ubiquitination

The 2 µg Ub-HA plasmid was transfected into cells stably expressing shNC and shENY2. After 72 h, the cells were treated with 10 µmol/L MG132 (YEASEN, China) for 6 h. The cleaved protein sample and 4 µg of p53 antibody were used for the experiment according to the co-ip protocol described above. Anti-HA antibody was used to detect ubiquitination.

### mRNA decay assay

Breast and colon cancer cell lines transfected with siNC and siENY2 were subsequently treated with actinomycin D (10 µg/mL) (Glpbio, USA) for 0, 12, 24, 36, and 48 h, respectively. RNA was extracted and endogenous IL11 mRNA levels were analyzed using qRT-PCR. The IL11/GAPDH ratio was calculated based on GAPDH levels.

### Statistical analysis

The statistical evaluation encompassed the utilization of GraphPad Prism 9.5 for both t-test and analysis of variance (ANOVA) to derive the p-value. Each in vitro experiment was conducted in triplicate. Statistical significance was determined by a p-value of less than 0.05, with the level of significance denoted as follows: * for *P* < 0.05, ** for *P* < 0.01, and *** for *P* < 0.001. Quantitative results were expressed as the mean accompanied by the standard deviation (SD).

## Results

### ENY2 exhibits significant overexpression across multiple tumor types and is closely linked to adverse clinicopathological characteristics and unfavorable patient outcomes

**Fig. 1:**
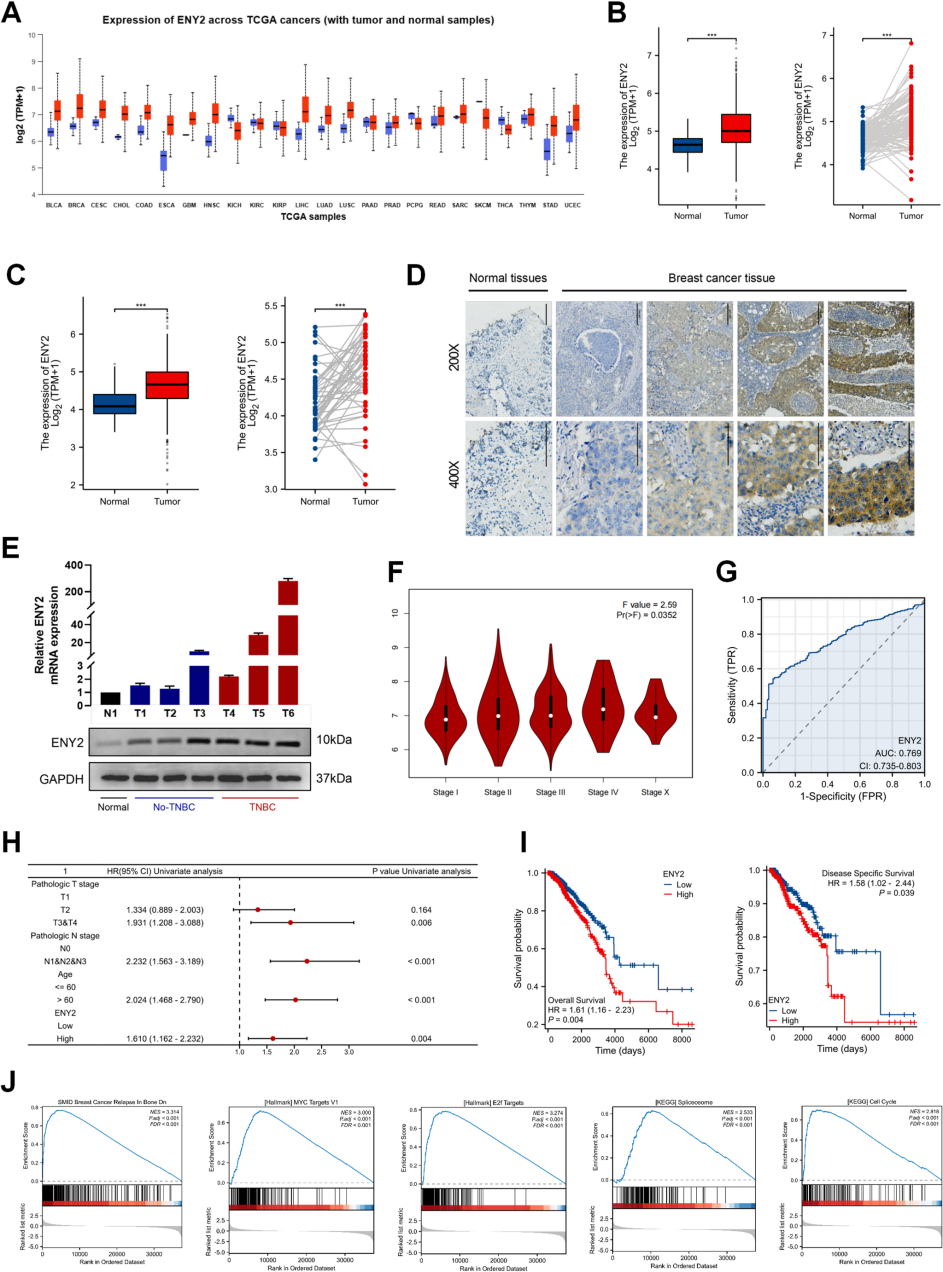
Identifies ENY2 as an oncogene. (**A**) Utilizing the UALCAN platform, a comprehensive analysis was conducted on the mRNA expression of ENY2 across various cancer and paracancerous tissues derived from the TCGA database. (**B**-**C**) The differential mRNA expression levels of ENY2 were scrutinized in both paired and unpaired breast and colon tissues sourced from the TCGA database. (**D**) Immunohistochemical staining was employed to assess the expression of ENY2 in adjacent normal breast tissues and multiple breast cancer tissues. The representative images of three independent experiments are shown. (**E**) qPCR and Western Blot techniques were utilized to evaluate the mRNA and protein levels of ENY2 in breast cell lines. (**F**) The GEPIA2.0 database was leveraged to investigate the association between ENY2 expression and the pathological stages of breast cancer. (**G**) The predictive efficacy of ENY2 on the survival outcomes of breast cancer patients was assessed using a ROC curve analysis. (**H**) Forest plots were generated to depict the prognostic significance of ENY2 expression in breast cancer patients. (**I**) Kaplan-Meier survival curves were constructed to illustrate the impact of ENY2 expression on the overall and disease-free survival rates of breast cancer patients. (**J**) GSEA was performed to elucidate the potential pathways implicated by ENY2 in breast cancer

To screen for genes related to the malignant biological behavior of tumors, we included four high-quality ChIP-seq datasets; ENY2 was screened as the target gene (Fig. S1A), and ENY2 was significantly amplified in human cancers (Fig. S1B). Using data from the TCGA and UALCAN platforms, we preliminarily analyzed the transcriptional and protein levels of ENY2 in different tumors. The results showed that ENY2 was expressed at high mRNA (Fig. 1A and S1C) and protein (Fig. S1D-1 F) levels in most tumors. These results suggest that ENY2 functions as an oncogene in tumors. Consequently, an in-depth examination was conducted to elucidate the expression of ENY2 within both cancer cells and their associated tissue environments. First, the transcript levels of ENY2 in paired and unpaired tumors and normal tissues were detected. Breast and colon cancer tissues showed higher ENY2 expression levels than adjacent normal tissues (Fig. 1B and C). The immunohistochemical evaluation revealed a marked upregulation of ENY2 in breast cancer tissues when compared to normal breast tissue samples (Fig. 1D). Additionally, qPCR and western blotting techniques were utilized to quantify the mRNA and protein levels of ENY2 in different breast cancer cell lines. The findings demonstrated a higher expression of both mRNA and protein for ENY2 in breast cancer cells, with the most significant enhancements observed in triple-negative breast cancer cell lines (Fig. 1E). MCF-7 and BT-549 cells exhibited higher protein and transcriptional levels of ENY2 and were therefore selected for subsequent functional experiments.

To investigate the relationship between ENY2 expression and the clinicopathological features of tumors, clinical tissue samples were categorized into groups based on high and low ENY2 expression levels. Statistical analysis was conducted using the chi-square test to assess these correlations. The findings indicated that in breast cancer, ENY2 expression levels were associated with the T stage and hormone receptor status, as detailed in Table 1. Furthermore, ENY2 expression was found to be linked to the clinicopathological stage of breast cancer (Fig. 1F). In colorectal cancer, ENY2 levels were correlated with lymph node involvement and pathological staging, with a higher proportion of patients exhibiting high ENY2 expression among those with positive lymph node metastasis. Additionally, a greater proportion of patients in pathological stages III–IV demonstrated high ENY2 expression, as summarized in Table 2.

In light of these findings, we conducted an examination of the correlation between ENY2 expression levels and patient survival outcomes. The ROC curve suggested that ENY2 could be used as a predictive discriminant factor in the prognostic analysis (Fig. 1G). A regression analysis conducted on the forest plot revealed that elevated ENY2 expression serves as a detrimental prognostic indicator for breast cancer (Fig. 1H). Utilizing the TCGA platform, we assessed the Kaplan-Meier survival curves for breast cancer patients, which indicated that individuals exhibiting high ENY2 expression experienced shorter Overall Survival and Disease Free Survival periods compared to those with lower ENY2 expression (Fig. 1I).

Cox regression analyses, both univariate and multivariate, were employed to investigate the relationship between the survival outcomes of breast cancer patients and the expression levels of ENY2, as well as various clinicopathological factors. The results showed that T stage, lymph node stage, patient age, and ENY2 expression levels could jointly affect the prognosis of patients with breast cancer (Table 3). Gene Set Enrichment Analysis (GSEA) revealed that ENY2 expression was significantly enriched in cell cycle and E2F target pathways during breast cancer progression (Fig. 1J), suggesting its potential involvement in cell cycle regulation.The above results indicate that high ENY2 expression in breast cancer has a certain significance in the evaluation of patient prognosis and has application value in clinical diagnosis and treatment.

**Table 1:** The relationship of ENY2 protein expression with the clinicopathological features of breast cancer patients

Characteristics	Low expression of ENY2	High expression of ENY2	*P* value
n	543	544	
Pathologic T stage, n (%)			**0.018**
T1	144 (26.5%)	134 (24.6%)	
T2	296 (54.5%)	335 (61.6%)	
T3	86 (15.8%)	54 (9.9%)	
T4	17 (3.1%)	18 (3.3%)	
PR status, n (%)			**< 0.001**
Negative	130 (25.1%)	212 (41.1%)	
Positive	388 (74.9%)	304 (58.9%)	
ER status, n (%)			**< 0.001**
Negative	74 (14.3%)	166 (32.0%)	
Positive	445 (85.7%)	352 (68.0%)	
HER2 status, n (%)			**0.023**
Negative	300 (81.5%)	260 (74.5%)	
Positive	68 (18.5%)	89 (25.5%)	

**Table 2:** The correlation between ENY2 protein expression and the clinicopathological features of colorectal cancer patients

Characteristics	Low expression of ENY2	High expression of ENY2	*P* value
n	322	322	
Pathologic N stage, n (%)			**0.032**
N0	198 (61.7%)	170 (53.3%)	
N1&N2	123 (38.3%)	149 (46.7%)	
Pathologic stage, n (%)			**0.017**
Stage I&Stage II	189 (60.8%)	160 (51.3%)	
Stage III&Stage IV	122 (39.2%)	152 (48.7%)	
Age, n (%)			**< 0.001**
<= 65	115 (35.7%)	161 (50%)	
> 65	207 (64.3%)	161 (50%)	

**Table 3:** Univariate and multivariate Cox regression analyses for overall survival in breast cancer patients

Characteristics	Total(*N*)	Univariate analysis		Multivariate analysis	
		Hazard ratio (95% CI)	*P* value	Hazard ratio (95% CI)	*P* value
Pathologic T stage	1,083				
T1	277	Reference		Reference	
T2	631	1.334 (0.889–2.003)	0.164	1.331 (0.848–2.088)	0.214
T3&T4	175	1.931 (1.208–3.088)	**0.006**	1.838 (1.089–3.101)	**0.023**
Pathologic N stage	1,067				
N0	516	Reference		Reference	
N1&N2&N3	551	2.232 (1.563–3.189)	**< 0.001**	2.206 (1.517–3.208)	**< 0.001**
Age	1,086				
<= 60	603	Reference		Reference	
> 60	483	2.024 (1.468–2.790)	**< 0.001**	2.473 (1.756–3.483)	**< 0.001**
ENY2	1,086				
Low	542	Reference		Reference	
High	544	1.610 (1.162–2.232)	**0.004**	1.790 (1.269–2.525)	**< 0.001**

### Knockdown of ENY2 inhibited tumor cell growth and proliferation

**Fig. 2:**
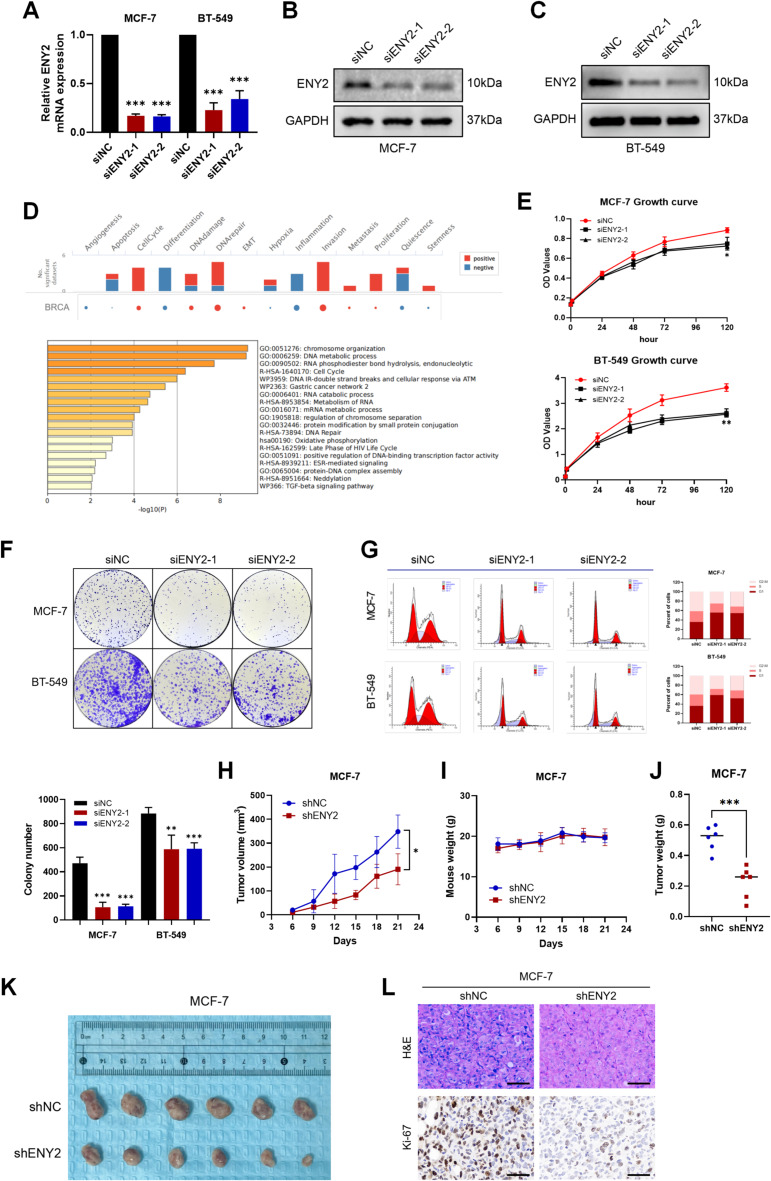
Knockdown of ENY2 inhibits the growth and cycle progression of breast cancer cells in vivo and in vitro. (**A**) Two types of siENY2 were transfected into two types of breast cancer cells carrying wild-type or mutant p53, and the knockdown efficiency was verified by qPCR after RNA extraction. (**B**-**C**) Two types of siENY2 were transfected into breast cancer cells, and knockdown efficiency was verified by western blotting. (**D**) The possible biological functions of ENY2 in breast cancer were analyzed using CancerSEA and Metascape. (**E**) CCK-8 assay was used to detect the effect of ENY2 knockdown on the proliferation of the two breast cancer cell lines. (**F**) To assess the impact of ENY2 gene suppression on the proliferative capacity of breast cancer cells, a plate-based colony formation assay was conducted. Data were analyzed using Student’ s t-test and are presented as the means ± SD. (**G**) Utilizing flow cytometry, alterations in the cell cycle progression were analyzed in the two breast cancer cell lines following the knockdown of ENY2 expression. (**H**-**I**) Tumor size and body weight of nude mice were detected every three days after orthotopic implantation of stably transfected control (shNC) and experimental (shENY2) MCF-7 cells in the breasts of nude mice. (J-K) Weight and size of the removed orthotopic tumors. (**L**) The growth of orthotopic tumors inoculated with MCF-7 cells transfected with shNC and shENY2 was examined by hematoxylin and eosin (H&E) and Ki-67 staining

Previous studies have suggested that ENY2 may function as an oncogenic factor, as it demonstrates elevated expression at both the protein and transcriptional levels in tumor tissues [29, 30]. Furthermore, ENY2 overexpression has been shown to promote invasion and lung metastasis in TNBC cells [31]. Consequently, our subsequent objective was to investigate its impact on the biological characteristics of tumors. We established two breast cancer cell lines (MCF-7 and BT-549 cell lines) through in vitro knockdown of ENY2. First, the knockdown efficiency of the two siRNAs was determined by qPCR and western blotting (Fig. 2A and C). Preliminary prediction of the biological function of ENY2 using the Metascape and CancerSEA databases suggested that ENY2 may be related to cell cycle and proliferation (Fig. 2D). Consequently, the CCK-8 assay was employed to evaluate cellular proliferation, revealing that the suppression of ENY2 hindered the proliferation of breast cancer cells (Fig. 2E). In parallel, the plate cloning assay demonstrated that the suppression of ENY2 also impaired the colony-forming capacity of breast cancer cells (Fig. 2F). The data indicated that breast cancer cell lines with reduced ENY2 expression exhibited a marked increase in G1 phase distribution and a corresponding decrease in S and G2-M phase distribution, suggesting that the suppression of ENY2 led to G1 phase cell cycle arrest. This observation was further corroborated by the finding that the knockdown of ENY2 resulted in G1 phase cell cycle arrest (Fig. 2G). Additionally, Transwell and cell scratch assays revealed that the migratory capacity of cells was diminished following ENY2 suppression (Fig. S2A-2 C). Consistently, transfection with shENY2 reduced the size and weight of xenograft tumors (Fig. 2H and J), Transfection efficiency was verified by WB, qPCR, and fluorescence (Fig. S3A-3 F), by constructing stable ENY2 knockdown MCF-7 cell lines for breast subcutaneous fat pad injection. In vivo, tumor proliferation and growth rates were significantly delayed (Fig. 2K and L).

Furthermore, we conducted functional studies by persistently enhancing ENY2 expression in the MCF-7 and the RKO cell line via lentiviral transduction. Conversely, the upregulation of ENY2 markedly accelerated the proliferation and colony formation of tumor cells (Fig. S4A-4 C). In a contrasting scenario, the overexpression of ENY2 significantly augmented cell proliferation and colony formation (Fig. S4D). These findings collectively indicate that the suppression of ENY2 expression curtails tumor cell growth and malignant proliferation both in vitro and in vivo.

### Knockdown of ENY2 inhibited 40 S and 60 S biosynthesis and induced nucleolar stress

**Fig. 3:**
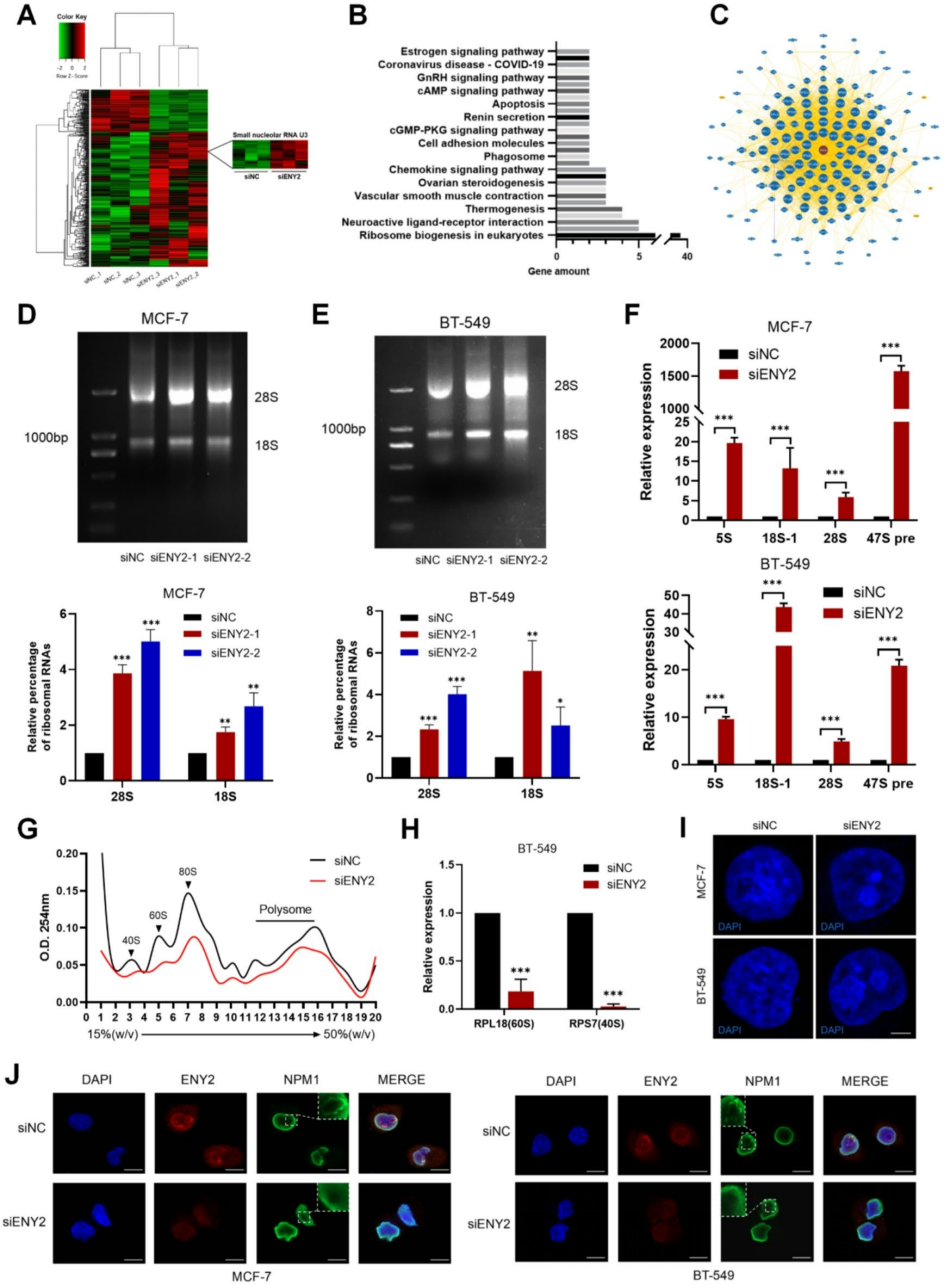
ENY2 is required for biosynthesis of 40 S and 60 S ribosomes. (**A**) RNA sequencing analysis shows the increased level of U3 snoRNA by heat map after knocking down ENY2. (**B**) Enriched signaling pathways revealed by analysis of differential genes transfected with siNC and siENY2. (**C**) The BioGRID platform shows the possible interactions of ENY2 with various proteins. (**D**-**E**) Assessment of rRNA concentrations via agarose gel electrophoresis subsequent to the transfection of two siENY2 constructs into MCF-7 and BT-549 breast cancer cell lines. Data were analyzed using Student’ s t-test. (**F**) Alterations in the levels of 5 S, 18 S, 28 S, and pre-47 S rRNA following the transfection of siENY2 into two breast cancer cell lines, as determined by qPCR analysis. (**G**) Ribosomal assay was performed to evaluate the biosynthesis of ribosomes after knockdown of ENY2, and the synthesis of 40 S, 60 S and 80 S ribosomes was detected by measuring the absorbance at 254 nm. (**H**) The mRNA levels of 40 S related protein RPS7 and 60 S related protein RPL18 after knocking down ENY2 were detected by qPCR. (**I**) DAPI staining showing morphological changes in nuclei and nucleoli after knocking down ENY2 in both breast cancer cells. Scale bar, 1 μm. (**J**) Immunofluorescence staining for ENY2 and NPM1 after transfection of siENY2 in two sheets of breast cancer cells. Representative images of three independent replicates are shown. Scale bar, 20 μm

The experimental results presented above demonstrate the oncogenic function of ENY2 in tumor biology. We hope to further explore the molecular mechanisms by which ENY2 promotes tumor development and progression. Transcriptome RNA sequencing (RNA-Seq) analysis of ENY2-knockdown tumor cells and heat map analysis of differentially expressed genes showed that the level of U3 small nucleolar RNA (snoRNA) significantly increased after ENY2 knockdown (Fig. 3A). At the same time, it was significantly enriched in the ribosome synthesis process of eukaryotic cells (Fig. 3B). SnoRNAs are mainly involved in post-transcriptional modification and maturation of rRNA, small nuclear RNA (snRNA), and other RNAs [47]. Previous studies have shown that U3 snoRNAs are required for nucleolar processing of pre-18 S ribosomal RNA [48, 49]. In addition, the BioGRID database suggested that ENY2 may interact with a variety of ribosomal proteins (RPs) (Fig. 3C). These findings indicate that ENY2 plays a role in the formation of ribosomes. The process of ribosome formation is intricate and involves multiple stages, including the transcription, the processing and the subsequent assembly of rRNA with ribosomal proteins to produce fully functional ribosomes [50, 51]. We further tested whether ENY2 affects ribosome biosynthesis. We transiently transfected siENY2 into MCF-7 and BT-549 cells and performed agarose gel experiments 48 h later. The qualitative results showed that after ENY2 knockdown, the levels of 18 S rRNA and 28 S rRNA increased to varying degrees (Fig. 3D and E). qPCR experiments also showed that ENY2 knockdown resulted in an increase in the levels of 47 S pre-RNA and the above rRNA (Fig. 3F). Tumor cells usually have a higher level of ribosome biogenesis to meet the needs of rapid proliferation and growth [52], and previous studies have shown that ENY2 knockdown could inhibit tumor growth.

The observed accumulation of precursor rRNA following ENY2 depletion suggested a potential defect in ribosome biogenesis. We therefore hypothesized that ENY2 is required for the efficient assembly or nuclear export of mature ribosomal subunits. To test this, we examined the abundance of mature 40 S, 60 S, and 80 S ribosomal subunits and analyzed polyribosome profiles by sucrose gradient centrifugation. ENY2 knockdown inhibited the production of 40 S, 60 S, and 80 S ribosomal subunits (Fig. 3G). The transcription levels of the 40 S related protein RPS7 and 60 S related protein RPL18 [53] were also significantly decreased (Fig. 3H).

The disruption of key phases within the ribosome biosynthesis pathway is a well-established inducer of nucleolar stress. This is robustly demonstrated by specific interventions, such as inhibition of RNA Polymerase I by Actinomycin D (Act.D) or genetic perturbation of nucleolar proteins Bop1 [54, 55], which impair ribosome production and trigger a nucleolar stress response characterized by nucleolar disruption and subsequent p53 activation [56]. Consequently, we sought to ascertain if nucleolar stress is induced following the suppression of ENY2. The presence of nucleolar stress can be detected through immunofluorescence analysis of nucleolar proteins, such as NPM1, which serves as a marker for nucleolar granule components [57, 58]. The morphology and size of nucleoli are intrinsically linked to their activity, which varies with cell type, growth, and metabolic state. In response to stress, nucleolar activity is altered, leading to characteristic morphological reorganizations [56, 59]. A hallmark of nucleolar stress is the condensation of these structures into smaller, more spherical shapes that resemble liquid droplets [5], with NPM1 relocating from the nucleolus to the nucleoplasm [60]. The immunofluorescence results revealed that DAPI staining demonstrated a significant condensation of the nucleolus into a globular form, and the suppression of ENY2 led to the translocation of NPM1 from the nucleolus to the nucleoplasm (Fig. 3I and J). These findings suggest that ENY2 deficiency is associated with reduced levels of 40 S and 60 S ribosomal subunits and can induce nucleolar stress.

### ENY2 interacts with nucleophosphoprotein to participate in ribosomal subunit export

**Fig. 4:**
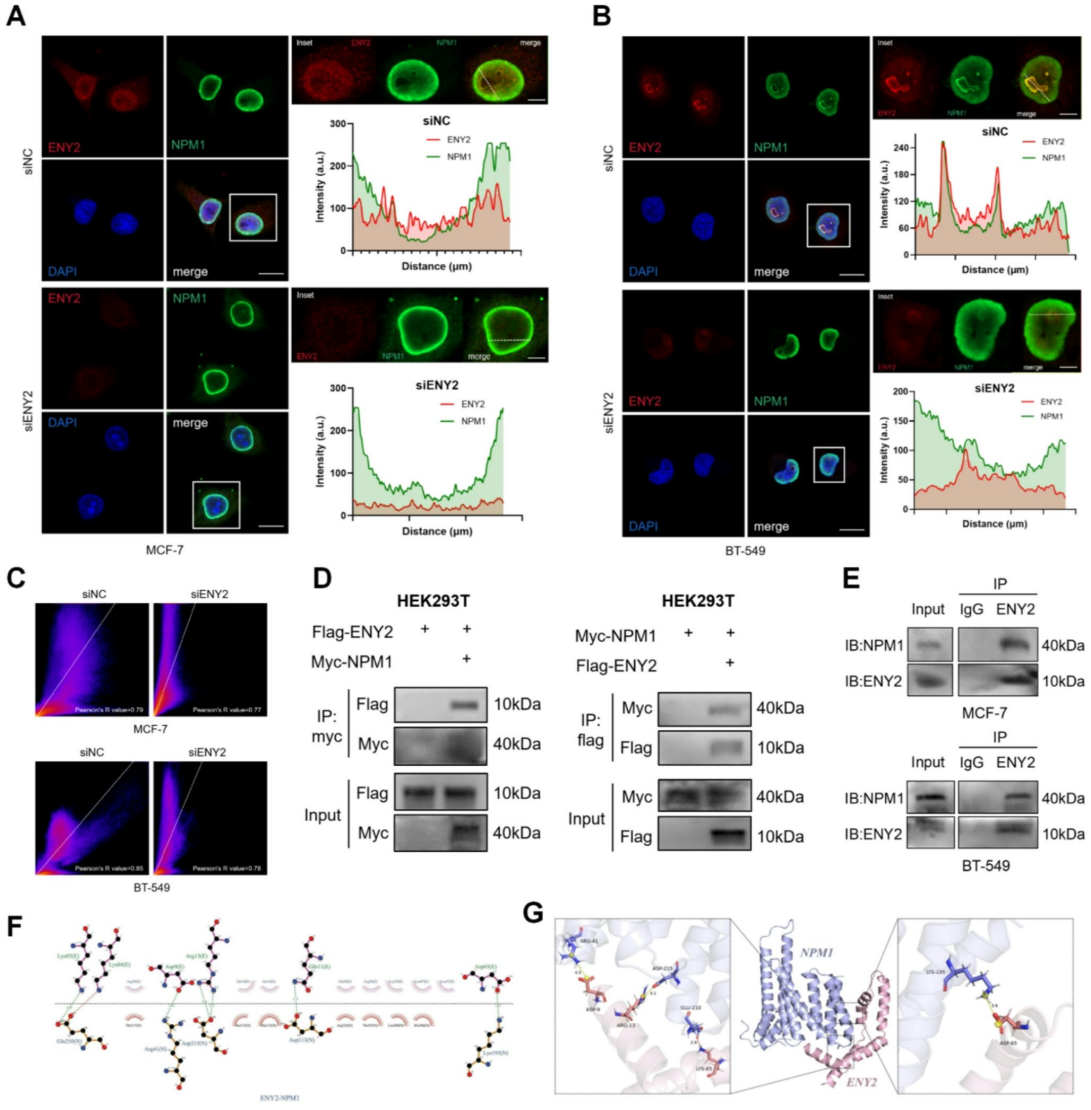
ENY2 co-acts on ribosome biosynthesis by binding to NPM1. (**A**-**B**) Immunofluorescence staining with anti-ENY2 (red) and anti-NPM1 (green) antibodies to show the co-localization of the two proteins after siENY2 transfection in MCF-7 and BT-549 cells. The nuclei were stained with DAPI. The signal intensity of each protein to the specified line was calculated by line plots. Scale bar, 20 μm (5 μm for insets). (**C**) The co-localization of ENY2 and NPM1 after knockdown of ENY2 in both breast cancer cells was analyzed using Image J. (**D**) Plasmids were transfected into 293T cells, and proteins were extracted for exogenous co-ip analysis. (**E**) Proteins were extracted from MCF-7 and BT-549 for endogenous co-ip assay. (**F**) 2D plots showing the protein interactions of ENY2 (E chain) and NPM1 (N chain) using Li plot software. The stick residues indicate the involvement of this residue in forming hydrogen bonds (green). The number near the dotted line of hydrogen bonds is the bond length in emmeters. (**G**) Visualization of the 3D positions of ENY2 (pink protein) and NPM1 (purple protein) by Pymol software. The red dashed line represents the hydrogen bond interaction, the yellow dashed line represents the salt bridge interaction, and the number near the dashed line of the interaction bond is the hydrogen bond length in emmeters

Having shown that ENY2 knockdown impairs 40 S and 60 S ribosomal subunit biogenesis, we next aimed to investigate the specific molecular mechanism underlying ENY2’s role in this process. Some studies have shown that NPM1 is mainly involved in the process of ribosome assembly and ejection [61, 62], we further explored the interaction between ENY2 and NPM1 using immunofluorescence co-localization and immunoprecipitation experiments. Immunofluorescence results showed that ENY2 and NPM1 were significantly co-localized in the nucleolus of breast cancer cell lines MCF-7 and BT-549 (Fig. 4A and B), and knockdown of ENY2 attenuated the co-localization. Pearson’s correlation coefficient (PCC) was calculated quantitatively using a scatter plot, and the results showed that knockdown of ENY2 also attenuated the strong co-localization relationship (Fig. 4C). The interaction between ENY2 and NPM1 was further verified by co-immunoprecipitation (Co-IP) experiments. The IP of exogenously expressed FLAG-tagged ENY2 and Myc-tagged NPM1 was performed using an endogenous complex (Fig. 4D and E), and the results confirmed the interaction between ENY2 and NPM1. To deepen our understanding of the interaction between ENY2 and NPM1, LigPlot software was used to draw 2D graphs to analyze bond formation between the two proteins (Fig. 4F), and PyMOL software was used for protein docking (Fig. 4G). Rosetta results showed that the binding free energy of ENY2 and NPM1 proteins was − 27.260 kcal/mol, indicating a large binding tendency. These results suggest that ENY2 interacts with NPM1 and is essential for ribosomal biosynthesis.

### ENY2 regulates p53 protein stability via the NPM1/MDM2 axis

**Fig. 5:**
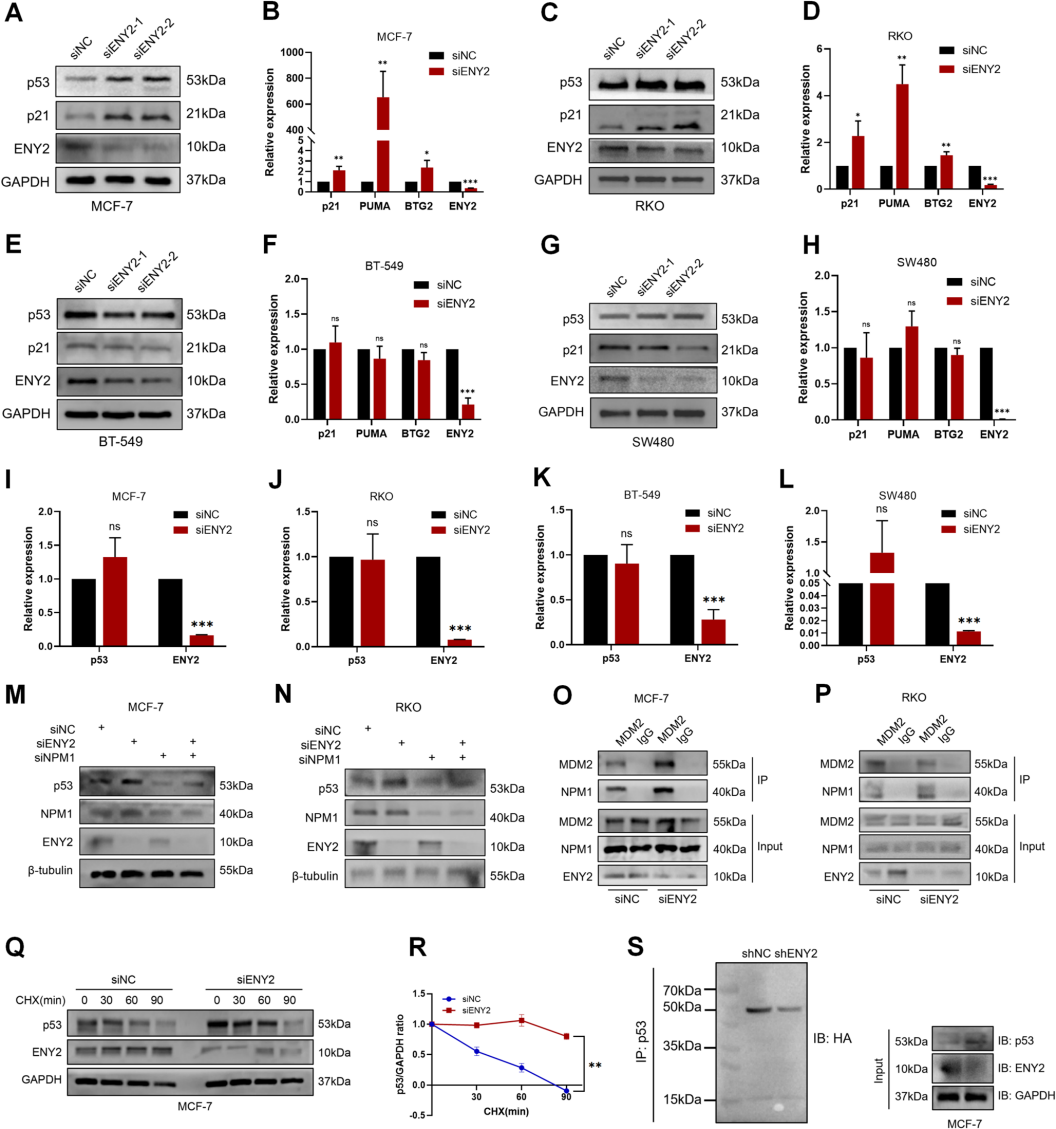
Knockdown of ENY2 inhibits the ubiquitination of p53 by MDM2 via NPM1. (**A**-**H**) Following the transfection of siENY2 into cell lines, protein and RNA were isolated. Subsequently, assess the levels of p53 protein and its downstream effector genes, namely p21, PUMA, and BTG2. (**I**-**L**) To investigate the impact of siENY2 on p53 transcription in breast and colon cancer cell lines. Data were analyzed using Student’ s t-test. (**M**-**N**) The expression of p53 protein was evaluated in MCF-7 and RKO cells post-transfection with siENY2 and siNPM1. (**O**-**P**) Co-ip was employed to examine the interaction between NPM1 and MDM2 in MCF-7 and RKO cells treated with MG132 following siENY2 transfection. (**Q**-**R**) In MCF-7 cells transfected with siENY2, the cells were collected after the addition of CHX to the culture medium for a specified duration, and the half-life of p53 was determined. GAPDH served as the internal control for normalization in the quantitative analysis. (**S**) The HA overexpression plasmid was transfected after knockdown of ENY2, and the p53 ubiquitination level was detected using anti-HA antibody

Defects in ribosome biogenesis normally lead to the accumulation and activation of p53 [63]. To delve deeper into the molecular pathways through which ENY2 facilitates tumorigenesis and progression, we conducted an analysis of the alterations in the expression of p53 and its downstream effector genes subsequent to ENY2 suppression. Our findings from Western blot and quantitative PCR analyses revealed that, following 72 h of siENY2 transfection in MCF-7 and RKO cell lines, there was a notable elevation in the protein levels of p53 and p21, accompanied by a corresponding increase in the mRNA levels of p53-regulated genes, including p21, PUMA, and BTG2 (Fig. 5A and D). Conversely, in BT-549 and SW480 cell lines, where ENY2 was downregulated, no substantial changes were observed in the protein levels of Mut p53 and p21, nor in the mRNA levels of the downstream target genes (Fig. 5E and H). The presence of a high incidence of p53 mutations in the triple-negative breast cancer cell line BT-549 and the colon cancer cell line SW480 suggests that the activation of p53 and its downstream targets following ENY2 knockdown is contingent upon the functional integrity of p53.

In addition, it is worth noting that changes in p53 mRNA content were detected by qPCR 48 h after knocking down ENY2, and we found that knocking down ENY2 did not affect the transcription of p53 (Fig. 5I and L), indicating that knocking down ENY2 mainly increased the level of p53 by changing the stability of the protein but did not affect the transcription level of p53.

Previous studies have found that after nucleolar stress, NPM1 interacts with MDM2 to inhibit the ubiquitination and degradation of p53 by MDM2, resulting in increased p53 activation and protein stability [64]. Based on our previous results, we found that ENY2 co-located and interacted with NPM1, and ENY2 knockdown caused nucleolar stress, translocating NPM1 from the nucleolus to the nucleoplasm and increasing p53 protein expression. Therefore, we tested whether ENY2 affects p53 protein levels via the NPM1-MDM2 axis. In MCF-7 and RKO cells, we examined whether NPM1 affected knockdown-induced change in p53 protein level induced by knockdown of ENY2. The results showed that The effect of ENY2 on p53 activation was weakened when NPM1 levels were inhibited (Fig. 5M and N). Next, we transfected siENY2 into tumor cells and performed co-immunoprecipitation experiments to examine the effect of ENY2 knockdown on the interaction between NPM1 and MDM2. The proteasome inhibitor MG132 was used to interrogate the ubiquitin-proteasome system. The finding that ENY2 depletion strengthens the interaction between NPM1 and MDM2 under MG132 treatment (Fig. 5O and P) provides a key mechanistic insight.

The observed increase in p53 protein stability upon ENY2 knockdown, as definitively shown by the cycloheximide (CHX) chase experiment (Fig. 5Q and R), prompted us to investigate the underlying degradation pathway. Additionally, a protein ubiquitination assay confirmed that ENY2 knockdown reduced p53 ubiquitination and increased p53 protein stability (Fig. 5S). We therefore propose that ENY2 normally acts to restrain the NPM1-MDM2 complex. Loss of ENY2 facilitates this interaction, thereby enhancing MDM2-mediated ubiquitination of p53 and targeting it for proteasomal degradation.

### Knockdown of ENY2 inhibited tumor growth and proliferation through p53-independent pathway

Previous studies have indicated that ENY2 functions to activate and maintain the stability of p53, thereby inhibiting tumor development and progression. To investigate the dependency of this effect on p53, we conducted experiments in breast cancer cell lines MCF-7 (which possesses wild-type p53) and BT-549 (which has a mutant p53). Our findings revealed that the suppression of ENY2 led to a reduction in cell proliferation and alterations in cell cycle dynamics in both cell lines. These observations imply that the regulation of tumor cell behavior by ENY2 knockdown occurs via a pathway that is independent of p53.

**Fig. 6:**
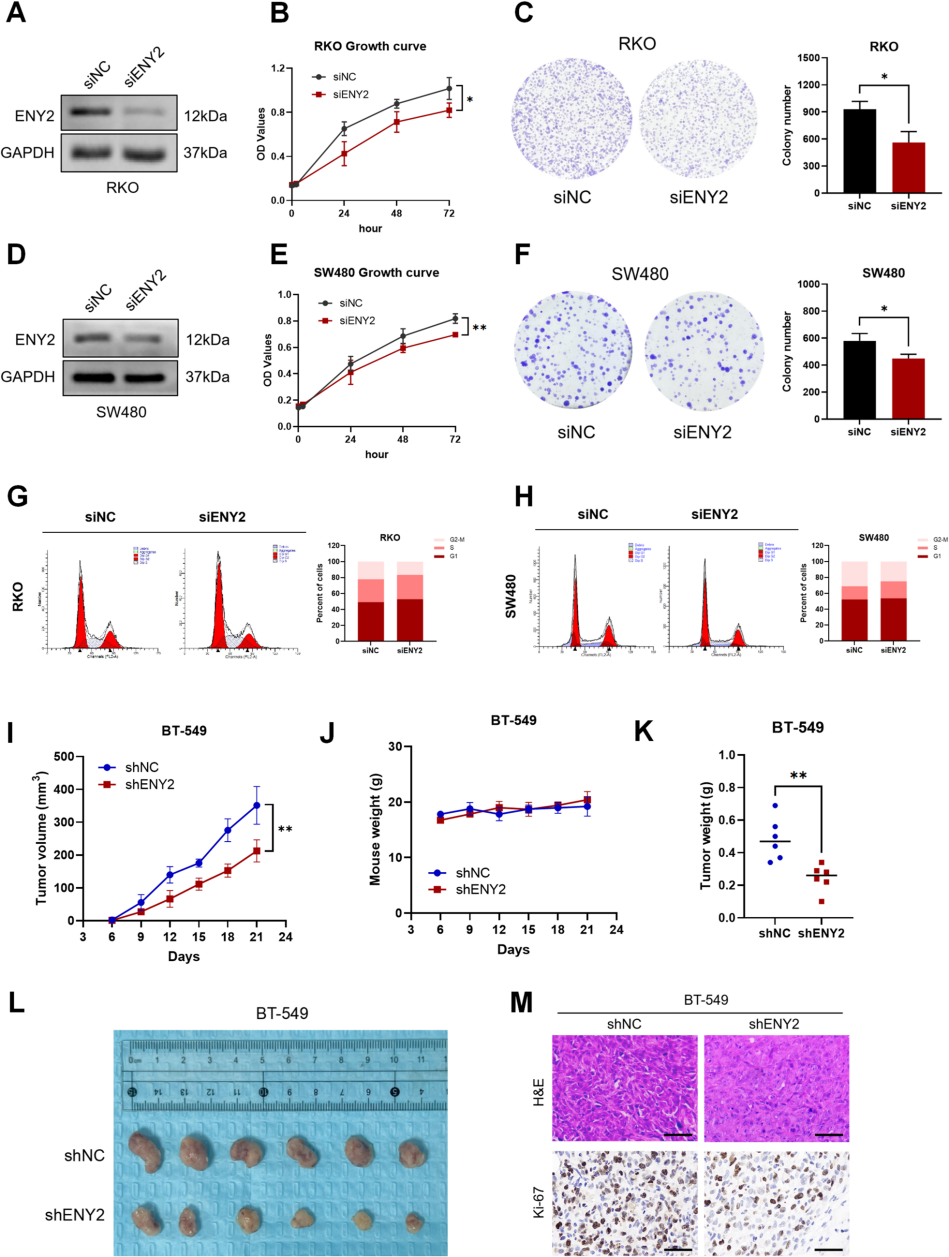
The tumor suppressive effect of ENY2 knockdown was partially p53-dependent (**A**-**F**) siENY2 was transfected into colon cancer cells respectively, and the protein was extracted to verify the knockdown efficiency by Western Blot. The effect of knocking down ENY2 in colon cancer cells on cell proliferation was detected by CCK-8 and plate cloning assay. (**G**-**H**) The changes in cell cycle distribution after knocking down ENY2 in colon cancer cells were detected by flow cytometry. (**I**-**J**) triple negative breast cancer cell BT-549 (mutant-type p53) from control (shNC) and stable ENY2 knockdown experimental group (shENY2) were orthotopic inoculated into the mammary gland of nude mice, and tumor size and body weight of nude mice were measured every three days. (**K**-**L**) The orthotopic tumors were removed to measure the tumor weight and size of the two groups. (**M**) Tumor growth of orthotopic tumors inoculated with BT-549 cells transfected with shNC and shENY2 was examined by H&E and Ki-67 staining

To further explore this, we employed colon cancer cell lines RKO (wild-type p53) and SW480 (mutant p53) and introduced siENY2. The results demonstrated that ENY2 depletion in RKO cells curtailed cell proliferation and colony formation (Fig. 6A and C). Notably, the knockdown of ENY2 in RKO cells resulted in an increased presence of cells in the G1 and S phases and a decreased presence in the G2 phase (Fig. 6G). In contrast, ENY2 knockdown in SW480 cells, while still inhibiting tumor cell proliferation (Fig. 6D and F), showed a more modest increase in the G1 phase population (Fig. 6H), suggesting a less pronounced effect compared to RKO cells.

In vivo studies also confirmed that the BT-549 cell line, after lentivirus-mediated shENY2 transfection (mutant p53), could be implanted into the breast fat pads of nude mice. The data indicated that low ENY2 expression hindered the proliferation and growth of orthotopic tumors (Fig. 6I and M). However, the in vivo suppression of tumor growth was less effective than the stable knockdown of ENY2 observed in MCF-7 cells.

Collectively, these results from breast and colon cancer cell models suggest that the suppression of ENY2 impedes tumor growth both in vitro and in vivo, operating through both p53-dependent and p53-independent pathways.

## Knockdown of ENY2 promoted IL-11 mRNA decay and pathway transduction through a RISC-mediated Silencing mechanism

**Fig. 7:**
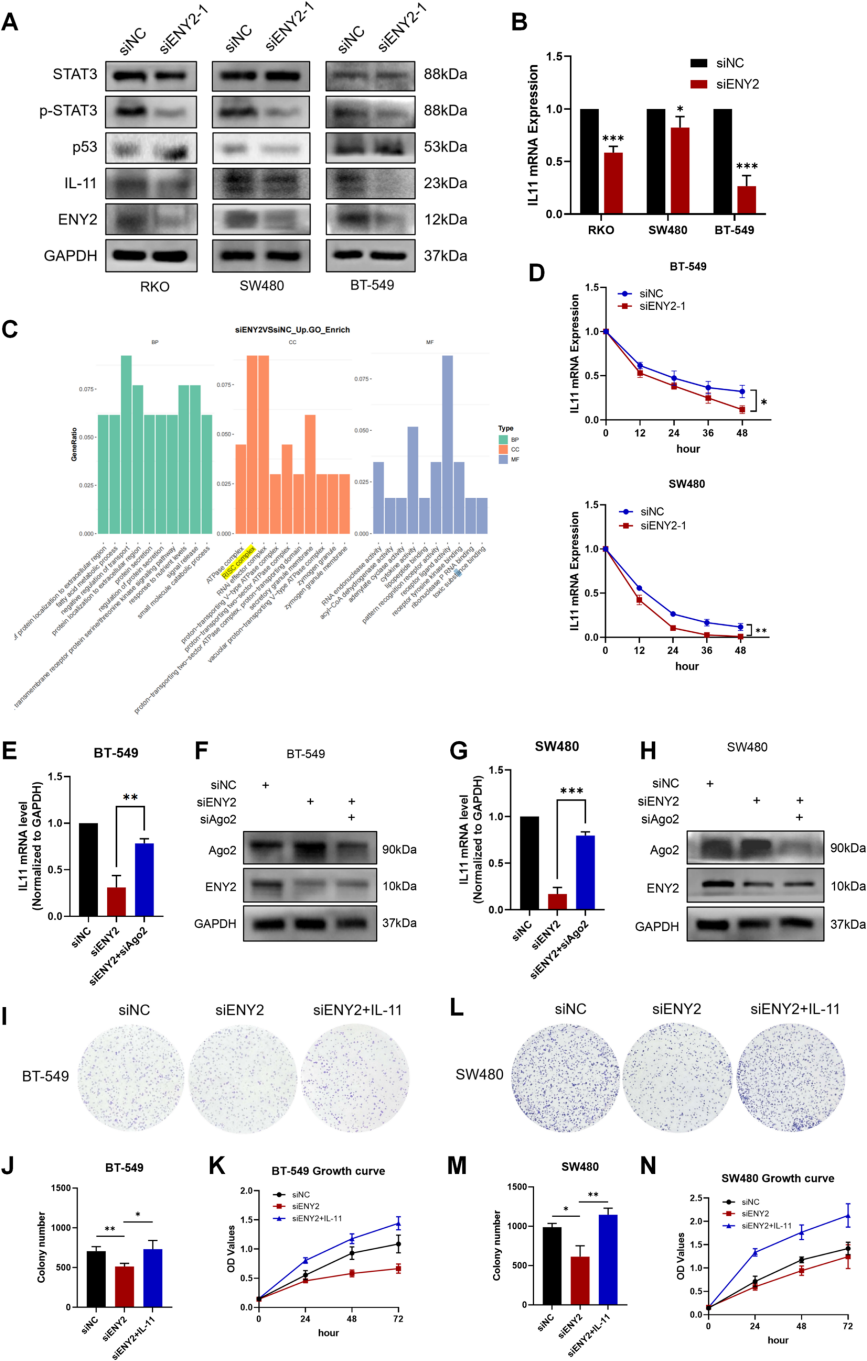
Knockdown of ENY2 promotes RISC-mediated IL-11 mRNA decay and inhibits signaling. (**A**) After knocking down ENY2, changes in the expression of IL-11, STAT3, and p-STAT3 were detected by WB. (**B**) qPCR was used to detect the mRNA expression level of IL11 after knocking down ENY2 knockdown in colon and breast cancer cells. (**C**) Sequencing analysis showed that ENY2 knockdown enriched GO terms for pathways and components with upregulated functions. (**D**) IL11 mRNA levels were measured by qPCR at each time point after actinomycin D treatment in both p53 mutant colon and breast cancer cells. (**E**-**H**) silencing of Ago2 reversed the mRNA decay of IL11 caused by ENY2 knockdown. The knockdown efficiency was verified by WB, and the expression of IL11 was detected by qPCR. (**I**-**N**) Addition of exogenous recombinant IL-11 to ENY2-depleted colon and breast cancer cells rescued tumor cell proliferation and colony formation ability

Given that ENY2 knockdown demonstrated a tumor-suppressive function independent of p53, we delved into the underlying molecular mechanisms. Our subsequent analysis of RNA sequencing data revealed that among the numerous differentially expressed genes, ENY2 knockdown notably diminished the expression of IL-11 (Fig. S5A). IL-11, a member of the glycoprotein-130 cytokine family, is implicated in the advancement of various malignancies, particularly those of epithelial origin, primarily through the activation of the JAK-STAT3 and PI3K-AKT-mTORC1 pathways [65]. Data from TCGA indicated that IL-11 exhibits elevated transcriptional levels in breast and colon cancer patients compared to normal tissues. Notably, while IL-11 expression is generally increased in breast cancer patients, it does not significantly vary between Luminal and TNBC subtypes. Furthermore, high IL-11 expression was not correlated with p53 mutation status (Fig. S5B-5 F). The cBioPortal database further corroborated a positive correlation between ENY2 and IL-11 expression in breast cancer patients (Fig. S5G). To substantiate these findings, we introduced siENY2 into colon cancer cell lines RKO and SW480, as well as the breast cancer cell line BT-549. qPCR and Western blot analyses demonstrated that ENY2 knockdown led to a reduction in both IL-11 mRNA and protein levels in RKO cells with wild-type p53 and p53-mutated SW480 cells, as well as in BT-549 cells (Fig. S5H). Additionally, phosphorylated STAT3 protein levels decreased, while total STAT3 levels remained unchanged (Fig. 7A and B). These observations collectively suggest that ENY2 knockdown attenuates IL-11 expression and JAK-STAT3 signaling activation, irrespective of p53 status.

We further explored the specific mechanism by which ENY2 knockdown inhibits IL-11 expression. Using an mRNA decay assay, we found that ENY2 knockdown promoted the decay of endogenous IL-11 mRNA (Fig. 7D). Based on the sequencing results, ENY2 knockdown was significantly enriched for an upregulated cellular component, the RNA-induced silencing complex (RISC complex) (Fig. 7C). Since RISC has been reported to target complementary Rnas in a directed manner via its bound microRNA (miRNA) and repress mRNA expression through cleavage, degradation, and translational repression [66], so we hypothesized that ENY2 knockdown may mediate IL-11 mRNA decay through a RISC-dependent mechanism. RISC generally functions through a small RNA and is a member of the AGO protein family, and we found that the ENY2-mediated decrease in IL-11 mRNA levels was reversed by Ago2 RNAi by siRNA transfection (Fig. 7E and H). To determine the role of IL-11 in ENY2-induced tumor growth, we added exogenous recombinant IL-11 to ENY2-knocked tumor cells for plate cloning and CCK-8 assays. The results showed that the addition of exogenous IL-11 rescued the proliferation inhibition caused by ENY2 knockdown (Fig. 7I and N). These results suggested that ENY2 knockdown promoted IL-11 mRNA decay via a RISC-mediated silencing mechanism.

### Targeting IL-11 overcomes ENY2-induced chemotherapy resistance in orthotopic xenografts of triple-negative breast cancer

**Fig. 8:**
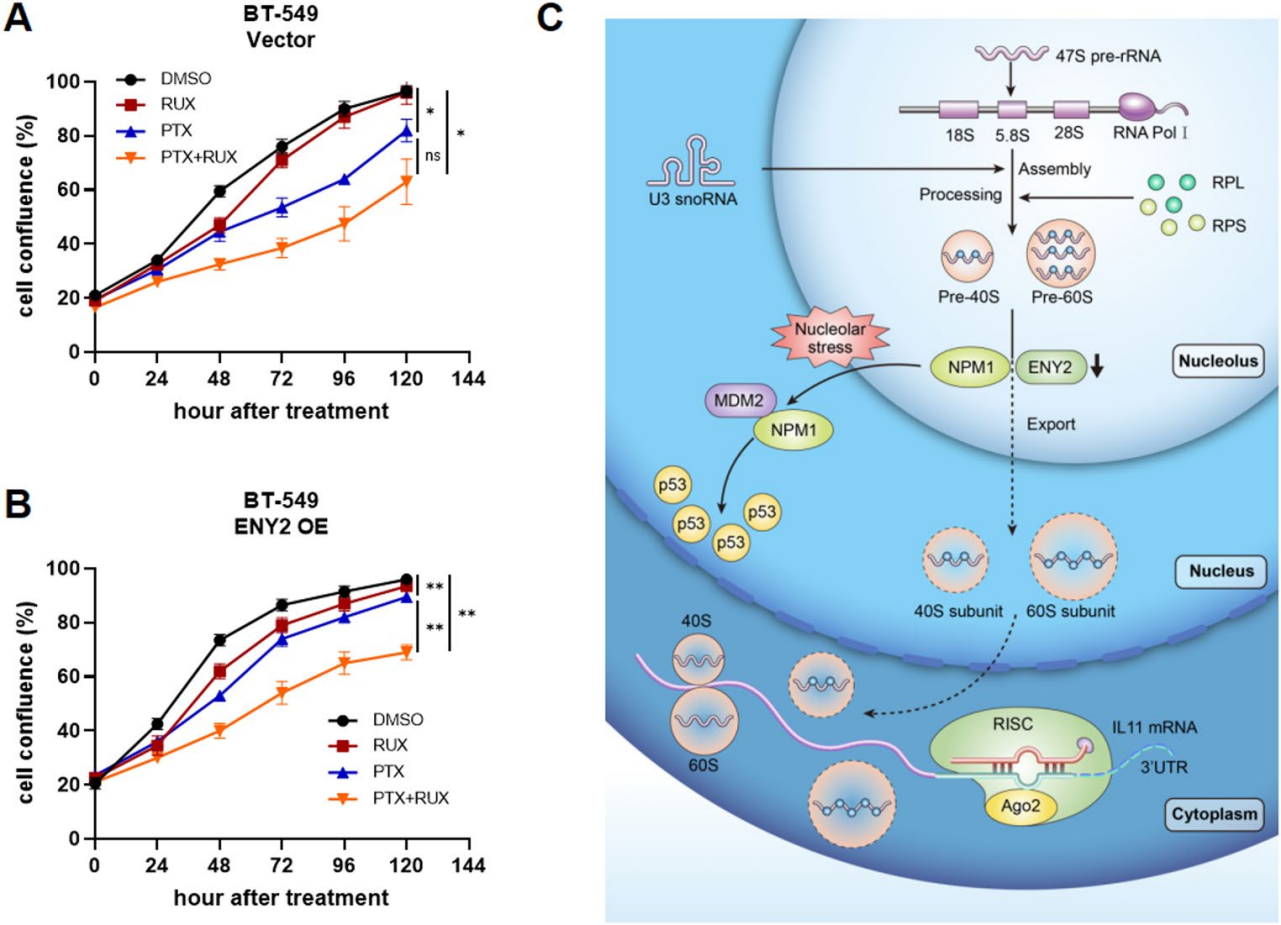
Combination of paclitaxel and anti-IL-11 downstream pathway therapy improves the treatment of ENY2-overexpressing tumors. (**A**-**B**) BT-549 cells with Vector and ENY2 OE treated with paclitaxel, resimertinib, paclitaxel and resimertinib, Cell growth was detected. (**C)** Schematic representation of ENY2 promoting tumor initiation and progression by inducing p53-dependent and independent nucleolar stress responses

To determine the role of ENY2 in tumor treatment and drug resistance, due to the lack of available drugs targeting the anti-IL-11 receptor, we chose to treat the downstream effects of IL-11 with ruoxlitinib (RUX), an inhibitor of JAK signaling pathway. In control BT-549 cells, treatment with 8 nM RUX did not induce any additional toxic effects (Fig. 8A). Surprisingly, we found that combined treatment with 1nM paclitaxel and 8 nM RUX in ENY2-overexpressing tumors reversed ENY2-induced paclitaxel resistance (Fig. 8B). These results indicated that targeting IL-11 signaling could restore ENY2-induced paclitaxel resistance. In conclusion, our study reveals that ENY2 deficiency inhibits tumor growth at both in vitro and in vivo levels by inducing nucleolar stress to activate p53-dependent pathways and RISC-mediated p53-independent pathways (Fig. 8C).

## Discussion

This study is significant because we identified the biological function and molecular mechanism of a novel oncogenic gene, ENY2, and revealed that RISC-mediated IL11 decay is a novel nucleolar stress downstream response outcome. ENY2 deficiency suppressed tumor growth in vitro and in vivo in a p53-dependent and-independent manner (Fig. 2), whereas ENY2 overexpression promoted tumor proliferation (Fig. S4). We have explained in detail the mechanism by which ENY2 interacts with NPM1 in the nucleolus to act together for ribosomal subunit export (Fig.s 3 and 4). ENY2 knockdown resulted in a decrease in the number of mature 40 S and 60 S subunits and a feedback increase in the levels of U3 snornas and several Rrnas, leading to a significant induction of nucleolar stress responses. In tumor cells expressing wild-type p53, we found that knockdown of ENY2 activated the classic p53-dependent nucleolar stress response and suppresses tumor cell progression by inhibiting p53 ubiquitination by MDM2. In most p53 mutant cells, the impairment of ribosomal subunit export caused by knockdown of ENY2 stimulates the binding and silencing efficiency of RISC to the target mRNA, thereby inhibiting IL-11 expression and the downstream JAK/STAT3 signaling pathway to suppress tumor proliferation, which is a novel p53-independent nucleolar stress response pathway. Combined treatment with JAK pathway inhibitors improved paclitaxel resistance caused by high ENY2 expression both in vitro and in vivo.

Overactivation of ribosome biogenesis plays a key role in the development and progression of cancer [67, 9]. Therefore, oncologists strategically regard inhibition of ribosome synthesis as a potential cancer treatment, and call the cellular stress response caused by defective ribosome synthesis as “nucleolar stress” or “ribosomal stress” [68]. Many processes can induce nucleolar stress, such as ultraviolet irradiation, heat shock, hypoxia, and application of chemotherapy drugs [69–72]. Compounds such as actinomycin D and 5-fluorouracil, which inhibit rRNA synthesis and processing [73, 74], and cisplatin and oxaliplatin, which effectively inhibit RNA Pol I transcription [75], have been used in cancer chemotherapy. Several seminal studies by Pestov and Rubbi [70, 76] have shown that nucleolar proteins and UV irradiation induce p53 activation and cell cycle arrest following nucleolar destruction. The main effector process is that after nucleolar destruction, several ribosomal proteins, such as Arf, NPM1 and RPL5/11, redistribute to the nucleoplasm [77–79] to bind to MDM2, thereby increasing the protein stability of p53 [80]. While staining for additional markers such as fibrillarin would have provided further morphological detail [56], the definitive response of NPM1 serves as a robust and validated signal for the induction of nucleolar stress in our model. Our results showed that ENY2 knockout in cancer cells with high metabolic levels induced nucleolar stress and enhanced the binding of NPM1 and MDM2 to promote p53 stability, leading to tumor cell cycle arrest and the inhibition (Figs. 3, 4 and 5). If this effect is absent in normal cells with a low requirement for ribosome synthesis, targeting ENY2 would be an exciting approach for cancer therapy.

However, more than 50% of human cancers lose the function of wild-type p53 [81], and how the cell cycle responds to nucleolar stress has become difficult to study. A limitation of our study is the use of a single TNBC model, BT-549, which carries the specific p53 R249S mutation [82]. Consequently, the generalizability of our results regarding the tumor-suppressive role of ENY2 knockdown across different p53 mutant contexts requires further investigation. Future studies should employ isogenic cell lines (e.g., p53-wild-type cells engineered to express R249S) and additional p53-mutant models like MDA-MB-231 (R280K) to determine the dependency of ENY2’s function on specific p53 mutations [83]. This will clarify the mechanistic interplay between ENY2 and mutant p53 signaling pathways. Interestingly, even though MDM2 expression is lacking in metazoans such as Caenorhabditis elegans and Drosophila [84] and yeast does not express characteristic p53 or MDM2 [85, 86], a p53-independent nucleolar stress-induced cell arrest response exists in this ancient organism. This implied that the p53-independent nucleolar stress pathway could be exploited to target p53-mutant human cancers. This hypothesis has been described in several studies, including the inhibition of RNA pol I transcription by silencing POLR1A and the downregulation of E2F dependent on RPL11 release, which prevents the proliferation of p53-deficient cancer cells [87]. Another study proposed a new p53-independent response based on the activation of NF-κB caused by TIF-IA degradation as a new nucleolar stress downstream result [88]. It remains to be determined whether the effect of ENY2 on ribosome biogenesis is direct, through its involvement in pre-rRNA processing, or indirect, through modulating the expression of ribosome-related genes, or a combination of both. Here, we propose an unexpected p53-independent pathway for the nucleolar stress response. U3 snoRNAs and rRNA are basic components of ribosome synthesis, which eventually synthesizes the 40 S and 60 S subunits. After the ribosome maturates, the 40 S subunit recognizes and binds to mRNA, and the 60 S subunit is responsible for the formation of peptide bonds, together forming a “protein synthesis factory” [89]. Our study found that the deletion of ENY2 leads to the export of the 40 S and 60 S subunits, which in turn leads to the accumulation of raw materials for ribosome synthesis. Deletion of ENY2 results in defective ribosome synthesis, which reduces the occupancy of ribosomal subunits in the mRNA chain, thereby increasing the efficiency of RISC silencing. The RISC is composed of small Rnas and AGO proteins that regulate mRNA cleavage, degradation, and translational repression [66]. It has been shown that both the amount and location of RISC binding to the target mRNA can affect the efficiency of silencing, and the region occupied by ribosomes can reduce the effect of miRNA [90, 91]. Our results support the theory that ENY2 knockdown promotes IL11 mRNA decay and inhibits downstream signaling pathways through a RISC-dependent silencing mechanism (Fig. 7). Further studies are needed to determine which mirnas are involved in and affect exogenous IL11 transcription in the 3’UTR and how knockdown of ENY2 specifically acts on the RISC complex. Further mechanistic insights, such as determining direct RNA binding, remain an important goal for future work. This is an undescribed nucleolar stress pathway based on ribosomal subunit export dysfunction, which has also been observed in p53 mutant cells, and may be an effective strategy to target nucleolar stress in the treatment of mutant p53 tumors.

Anti-il-11 therapy prolongs the lifespan of mice of both sexes [92] and IL-11 plays a key role in tumorigenesis and immune escape [93]. Previous studies have described the role of IL11 in the activation of upstream and downstream pathways in the induction of paclitaxel resistance [94], and the differential efficacy of ruxolitinib, an inhibitor targeting IL11 downstream of JAK1/2, in patients with TNBC may be related to tumor heterogeneity [95]. Our results confirmed that in TNBC subtypes with high ENY2 and IL11 expression, the combination of ruxolitinib and paclitaxel has better efficacy than monotherapy. In addition, it has been shown that HEGBC gene binds to the promoter of IL-11, increases IL-11 transcription, and activates the downstream JAK/STAT3 signaling pathway. STAT3 binds to HEGBC promoter and activates its expression. HEGBC/IL-11/STAT3 promotes tumorigenesis by forming a positive feedback loop in tumors [96]. We hypothesized that STAT3 may bind to the promoter region of ENY2 and promote the high expression of ENY2 in TNBC, which requires further experimental exploration.

In conclusion, our data revealed that high ENY2 expression was a poor prognostic factor in patients with breast and colon cancers. ENY2 deficiency leads to impaired interactions with NPM1 and impaired export of ribosomal subunits. Eny2 deficiency activates p53-dependent cell growth arrest by inducing nucleolar stress and mediates RISC-dependent IL11 mRNA decay independent of p53 signaling. We provide insights into the targeted induction of nucleolar stress to suppress tumor progression through p53-dependent and p53-independent pathways, and the combination of anti-IL11 therapy in TNBC subtypes with high ENY2 expression may be a potential chemotherapy-sensitizing strategy.

## Supplementary Information

Below is the link to the electronic supplementary material.

Supplementary Material 1

Supplementary Material 2

Supplementary Material 3

## Data Availability

No datasets were generated or analysed during the current study.

## Abbreviations

rDNA: Ribosomal DNA
GC: Granular components
TNBC: Triple-negative breast cancer
Pol I: Polymerase I
ENY2: Enhancer of yellow 2 homolog
SAGA: Spt-Ada-GCN5-Acetyltransferase
H2Bub: H2B mono-ubiquitination
NPM1: Nucleophosmin
DMEM: Dulbecco’s modified Eagle’s medium
TCGA: The Cancer Genome Atlas
IHC: Immunohistochemical experiments
SD: Standard deviation
RNA-Seq: RNA sequencing
snoRNA: Small nucleolar RNA
snRNA: Small nuclear RNA
RPs: Ribosomal proteins
qPCR: Quantitative PCR
PCC: Pearson’s correlation coefficient
Co-IP: Co-immunoprecipitation
CHX: Cycloheximide
RUX: Ruoxlitinib

## Declarations

### Research involving Animals.

The animal experimental protocol was approved by the Experimental Animal Ethics Committee of Southern Medical University (SMUL2023011), and the study followed international ethical guidelines for animal experiments.
