# Supplementary material for: ENY2 transcription and export complex 2 subunit deficiency induces nucleolar stress to inhibit tumor progression through NPM1/MDM2/p53-dependent and -independent responses
Source: Cell Oncol (Dordr). 2026 Feb 5;49(1):41. doi: 10.1007/s13402-025-01148-4 (PMC12876109; doi:10.1007/s13402-025-01148-4)

Figure1E

ENY2

GAPDH

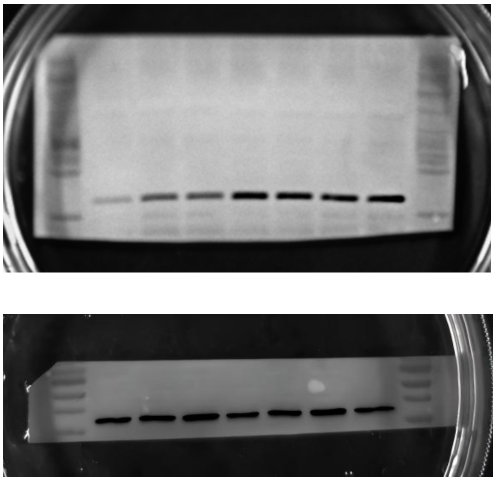

Figure2B

ENY2

GAPDH

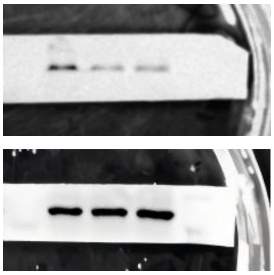

Figure2C

ENY2

GAPDH

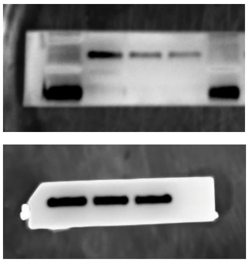

Figure3

MCF-7

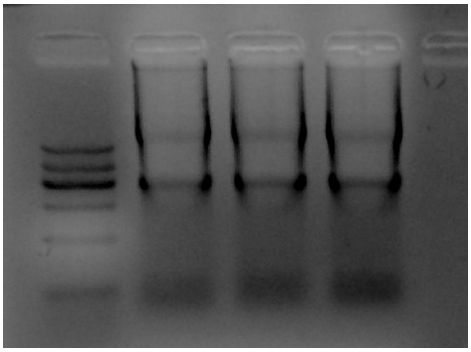

BT-549

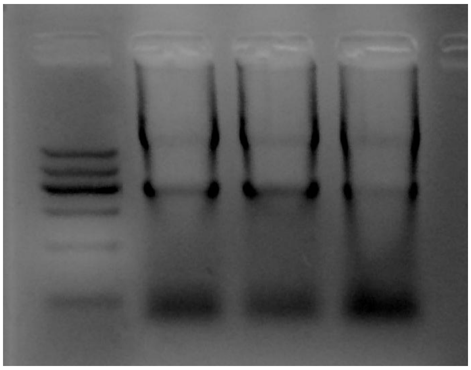

Figure4D

Flag

Myc

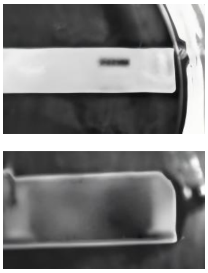

Myc

Flag

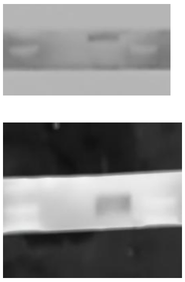

input:

Flag

Myc

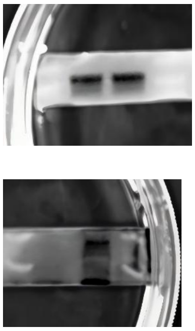

input:

Myc

Flag

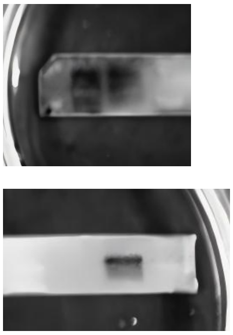

Figure4E

MCF-7

NPM1

ENY2

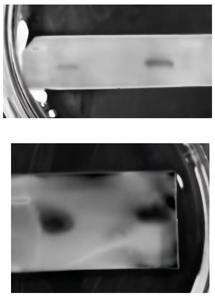

BT-549

NPM1

ENY2

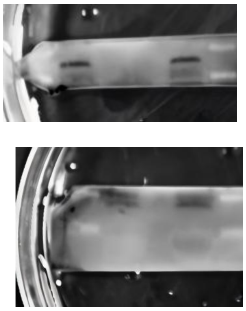

Figure5A

p53

p21

ENY2

GAPDH

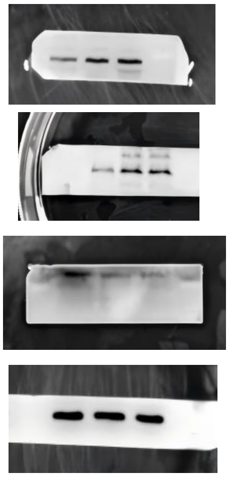

Figure5C

p53

p21

ENY2

GAPDH

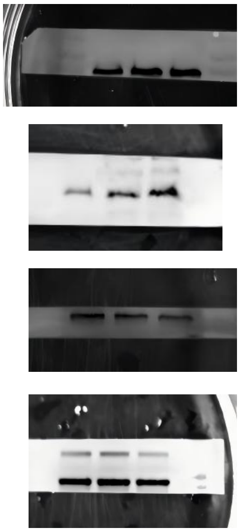

Figure5E

p53

p21

ENY2

GAPDH

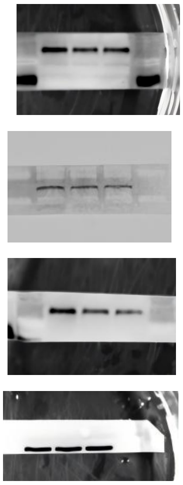

Figure5G

p53

p21

ENY2

GAPDH

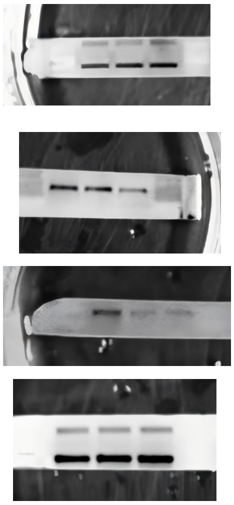

Figure5M

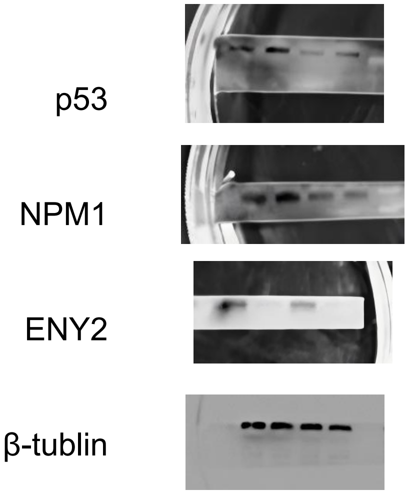

Figure5N

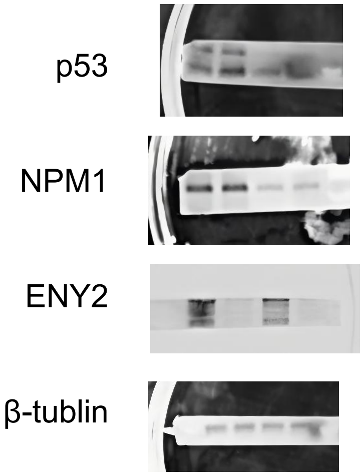

Figure5O

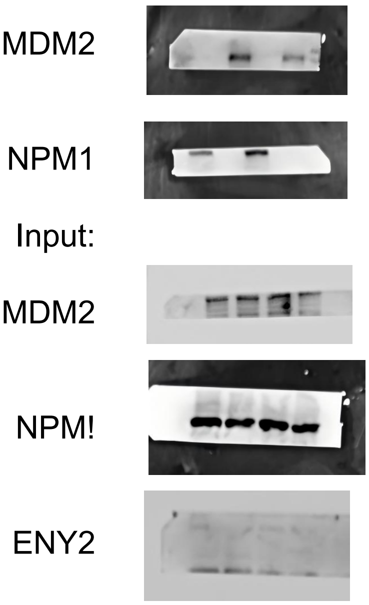

Figure5P

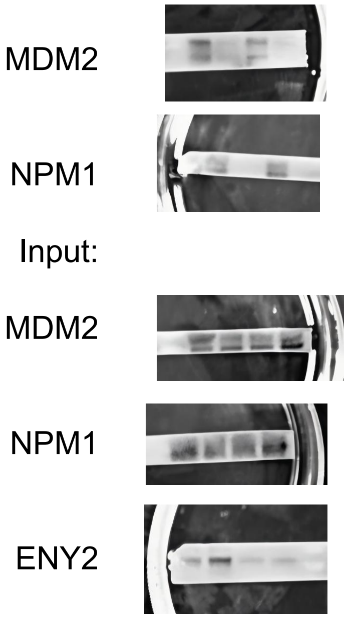

Figure5Q

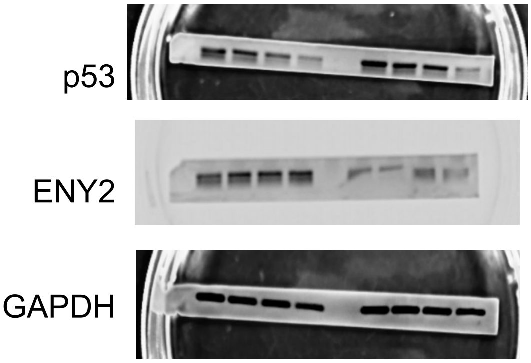

Figure5S

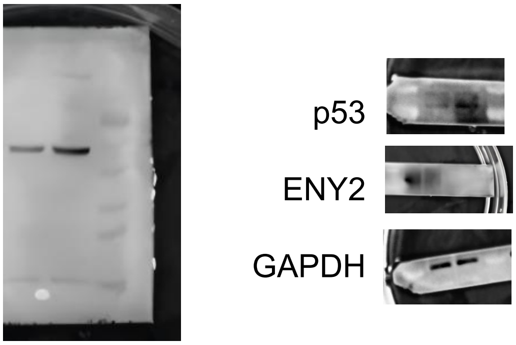

Figure6A

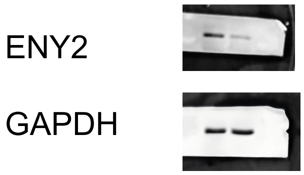

Figure6D

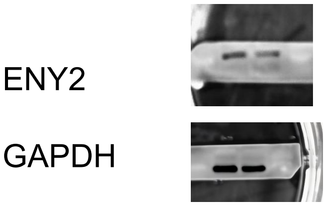

Figure7A

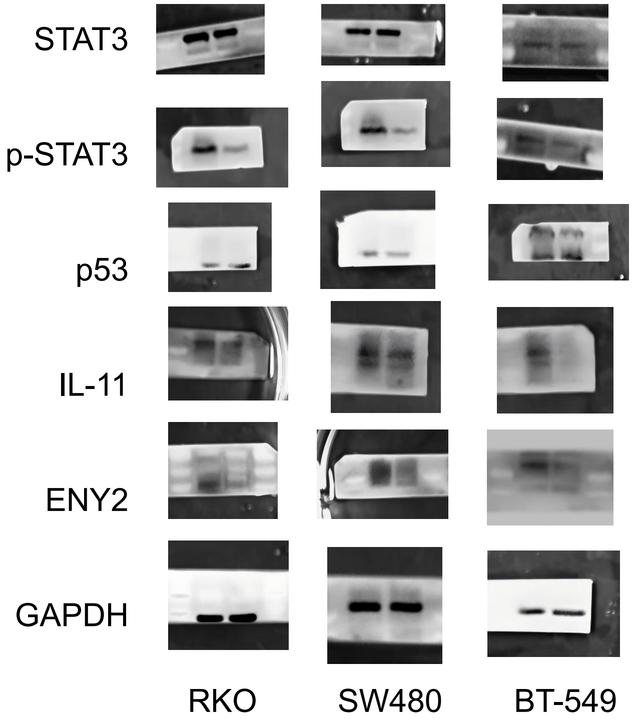

Figure7F

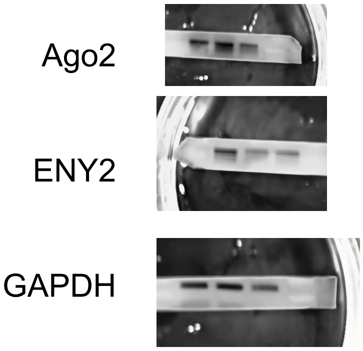

Figure7H

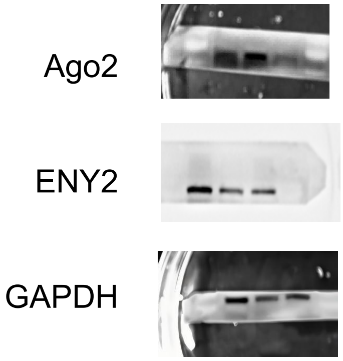

Supplement: Supplementary file 2 — Supplementary Material 2 [file 13402_2025_1148_MOESM2_ESM.pdf]
