# Supplementary material for: ENY2 transcription and export complex 2 subunit deficiency induces nucleolar stress to inhibit tumor progression through NPM1/MDM2/p53-dependent and -independent responses
Source: Cell Oncol (Dordr). 2026 Feb 5;49(1):41. doi: 10.1007/s13402-025-01148-4 (PMC12876109; doi:10.1007/s13402-025-01148-4)
Supplement: Supplementary file 3 — Supplementary Material 3 [file 13402_2025_1148_MOESM3_ESM.doc]

Supplementary materials


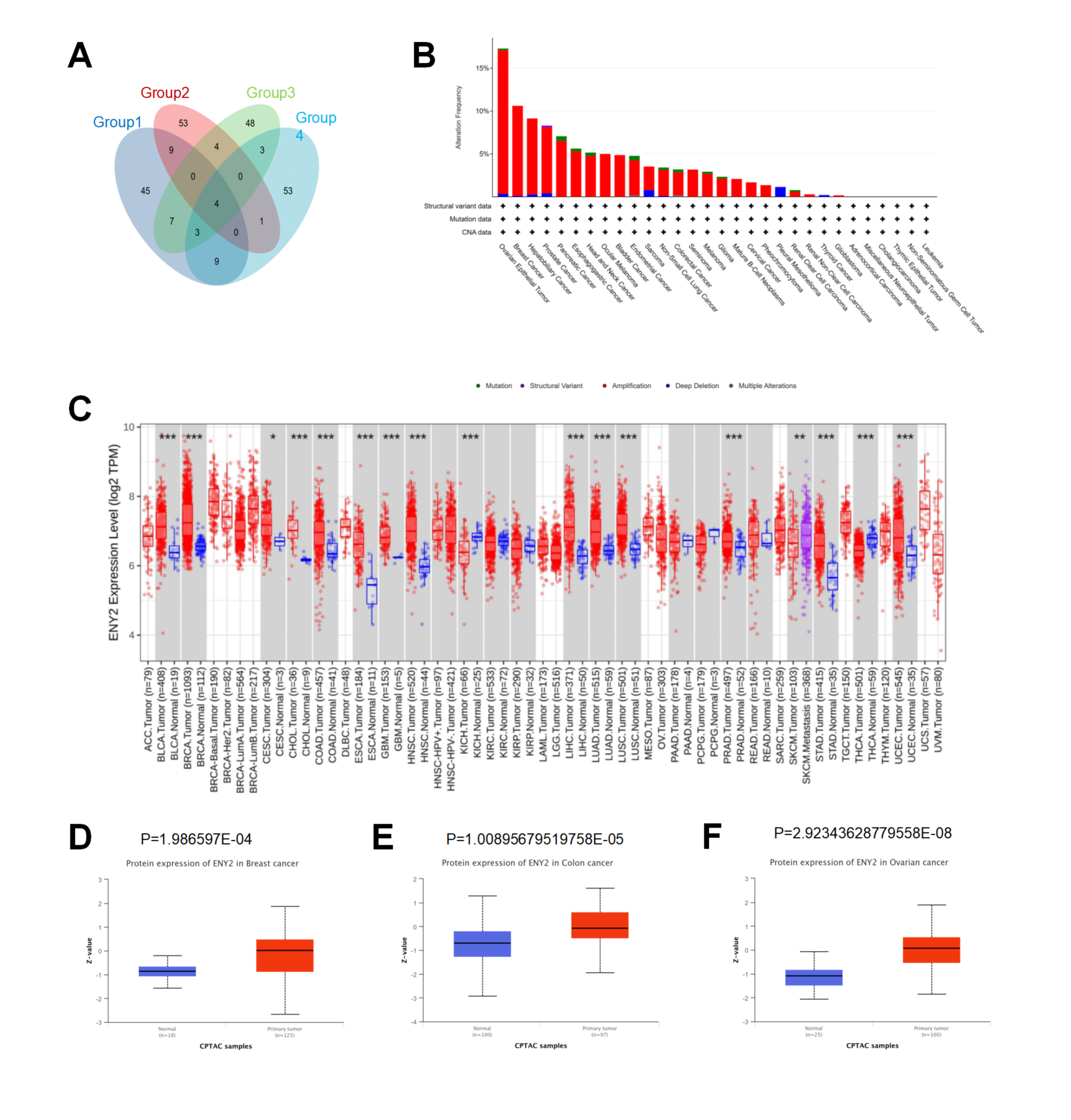


Figure S1. High expression of ENY2 in a variety of cancers

(A) Genes related to the malignant behavior of tumors were screened using Venn diagram analysis.

(B) The TCGA database shows that ENY2 is amplified in a variety of cancers.

(C) The GEPIA database shows that ENY2 expression in most tumors is higher than that in adjacent normal tissues.

(D-F) Differences in ENY2 protein expression in breast, colon, ovarian, and normal tissues.


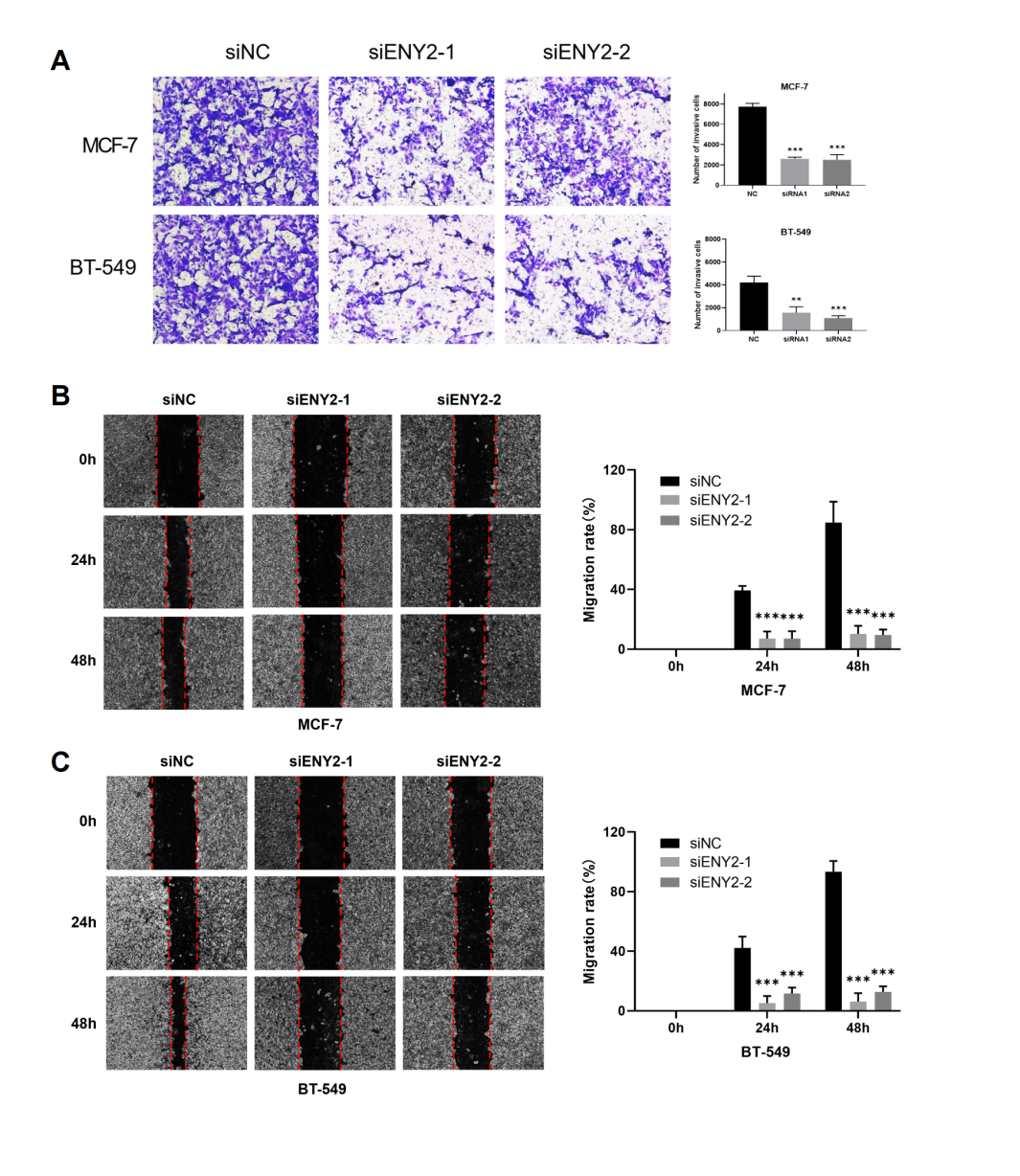


Figure S2. Knockdown of ENY2 inhibited the migration ability of breast cancer cells

(A) Transwell assays were performed to detect the migration ability of MCF-7 and BT-549 cells after ENY2 knockdown, and the number of migrating cells was quantitatively measured.

(B-C) Wound healing assay was used to analyze the migration of cells after ENY2 knockdown in the two breast cancer cell lines.


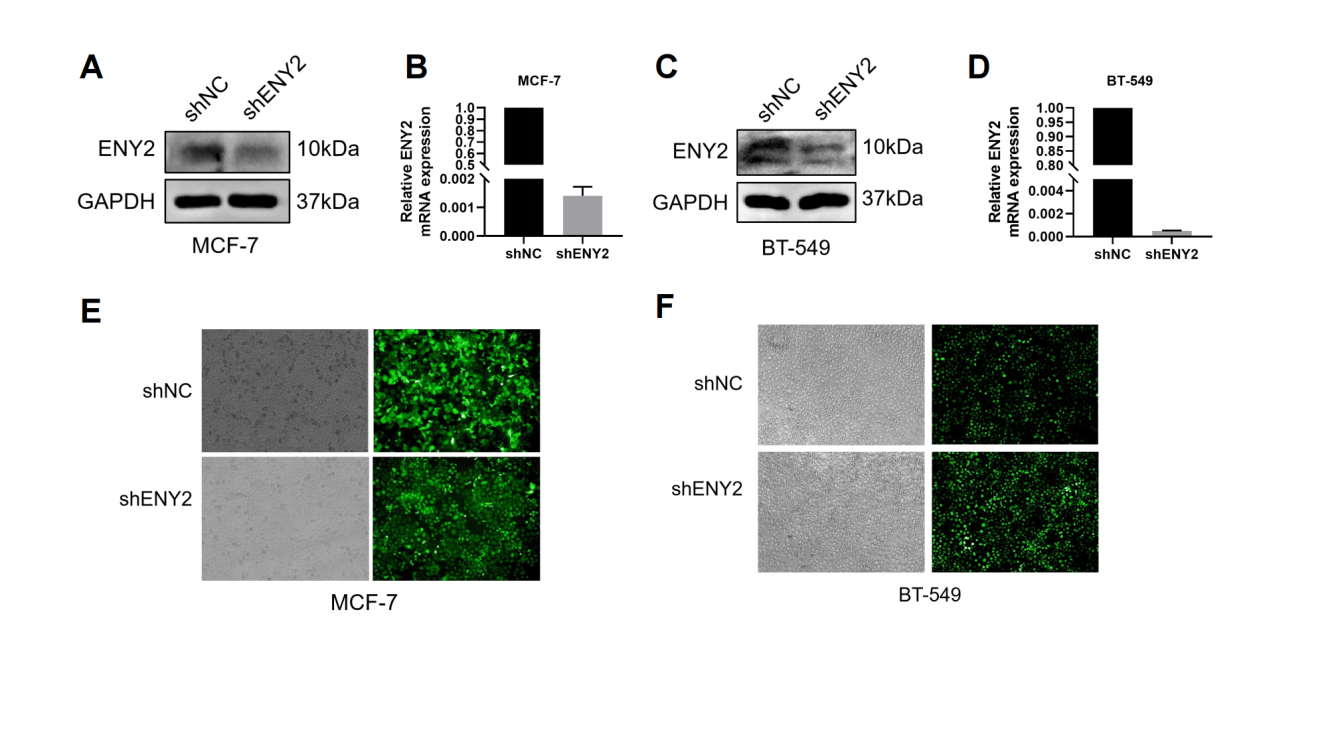
Figure S3. Validation of stable infection of ENY2 knockdown lentivirus in breast cancer cells

(A, C) Expression of ENY2 after stable lentiviral infection in MCF-7 and BT-549 cells was detected by WB.

(B, D) mRNA levels of ENY2 in MCF-7 and BT-549 cells after stabilization, as determined by qPCR.

(E-F) Microscopy and fluorescence visualization of transfection efficiency in cells three days after infection with lentivirus.


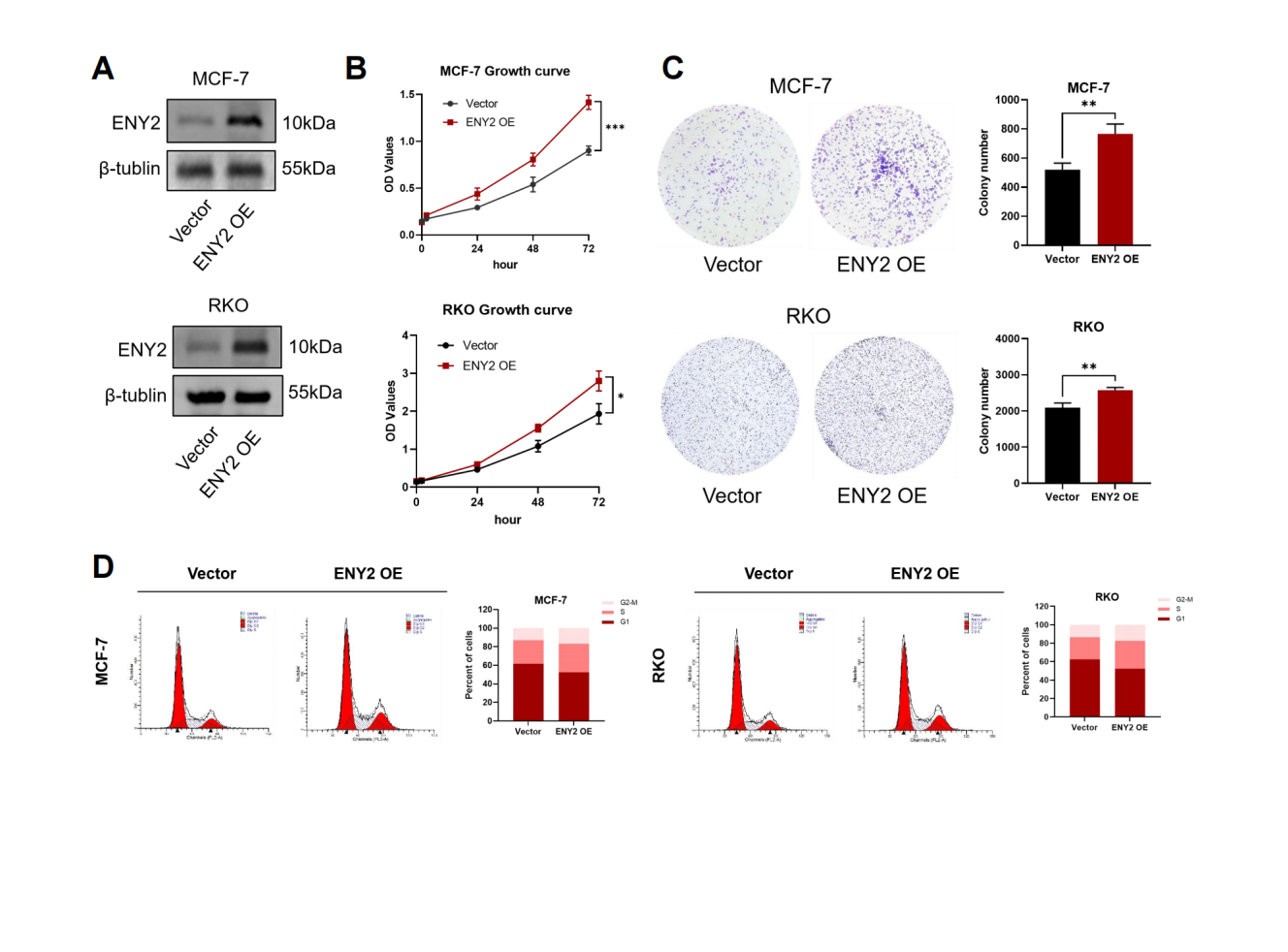
Figure S4. Overexpression of ENY2 promotes cell proliferation and cycle progression in breast and colon cancer cells

(A) ENY2 was overexpressed in breast and colon cancer cell lines expressing wild-type p53, and the expression of ENY2 was detected using western blotting.

(B) CCK-8 assay was used to detect the effect of ENY2 overexpression on the proliferation of breast and colon cancer cells.

(C) Plate cloning assay was used to detect the effect of ENY2 overexpression on the colony formation ability of breast and colon cancer cells.

(D) Flow cytometry was used to detect changes in cell cycle distribution after ENY2 overexpression in breast and colon cancer cells.


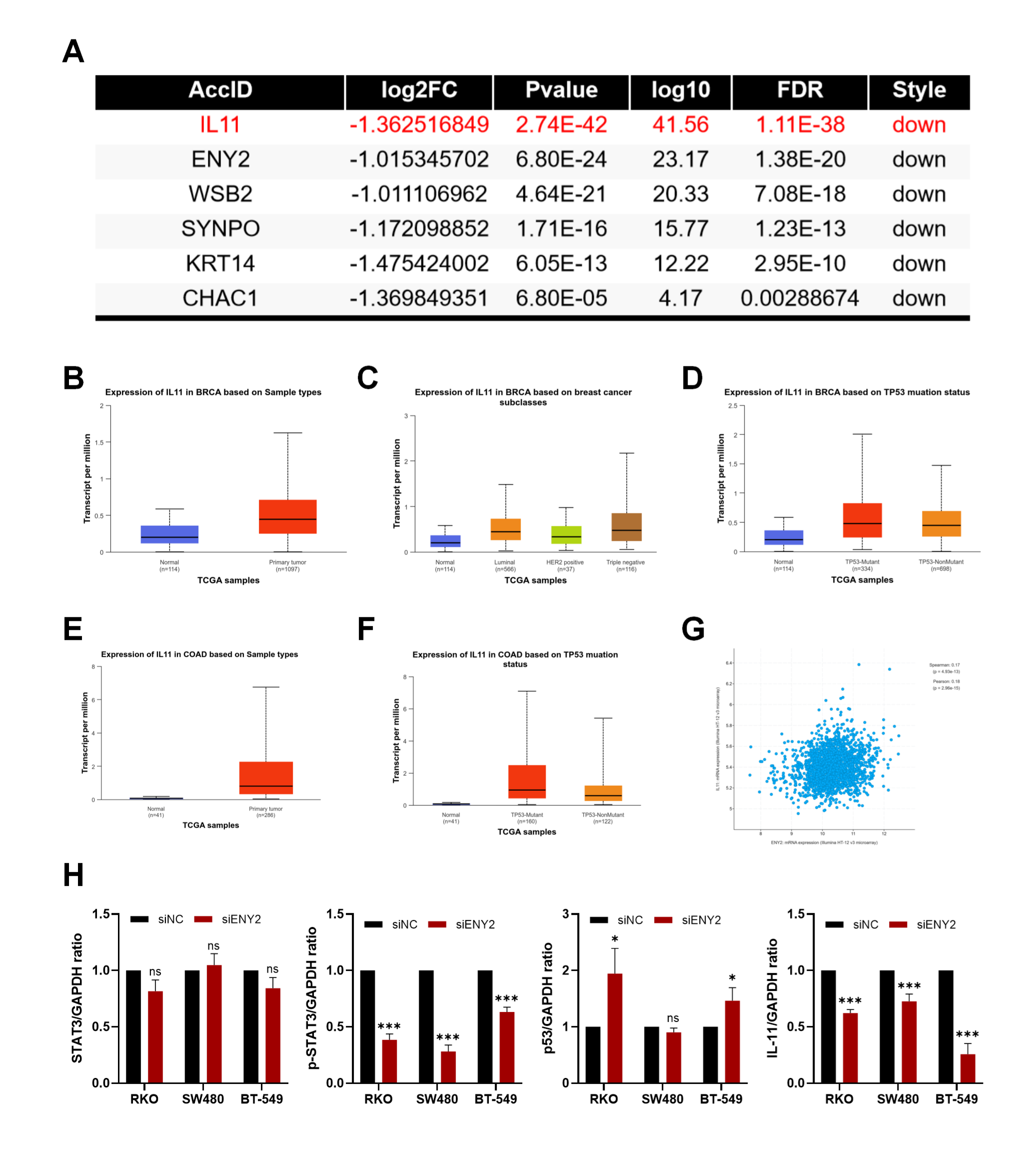


Figure S5. Knockdown of ENY2 inhibited IL-11 expression

(A) RNA-seq results showing the top6 differential gene expression after ENY2 knockdown.

(B) TCGA database shows that the transcriptional level of IL-11 in breast cancer tissues was higher than that in normal tissues.

(C) Expression of IL-11 in different breast cancer types; the expression level of IL-11 in TNBC was higher than that in the luminal subtype.

(D) Differential expression of IL-11 in breast cancer cells with different p53 mutation status.

(E) IL-11 expression is higher in colon cancer tissues than in normal tissues.

(F) Differential expression of IL-11 in colon cancer cells with different p53 mutation status.

(G) Positive correlation between ENY2 and IL-11 expression in breast cancer.

(H) Comparison of quantitative detection results of WB in Figure 7A.

Supplementary Table 1. Primers in the study.

| Primers used for quantitative RT-PCR | | |
| --- | --- | --- |
| Name | Forward (5’-3’) | Reverse (5’-3’) |
| GAPDH | 5'-TCACCACCATGGAGAAGGC-3' | 5'-GCTAAGCAGTTGGTGGTGCA-3' |
| ENY2 | 5'-TGAGAGCAGCATTAACCAAAA-3' | 5'-AGTGTGCCTTCAACTGATCCT-3' |
| 5S | 5'-GGCCATACCACCCTGAACGC-3' | 5'-CAGCACCCGGTATTCCCAGG-3' |
| 18S | 5'-CGCTTCCTTACCTGGTTGAT-3' | 5'-GAGCGACCAAAGGAACCATA-3' |
| 28S | 5'-AGAGGTCTTGGGGCCGAAACGATCTCAACC-3' | 5'-CTGATGAGCGTCGGCATCGGGCGCCTTAAC-3' |
| Pre-47S | 5'-GCTGACACGCTGTCCTCTGG-3' | 5'-ACGCGCGAGAGAACAGCAG-3' |
| NPM1 | 5'-ACGGTCAGTTTAGGGGCTG-3' | 5'-CTGTGGAACCTTGCTACCACC-3' |
| p53 | 5'-GAGGTTGGCTCTGACTGTACC-3' | 5'-TCCGTCCCAGTAGATTACCAC-3' |
| p21 | 5'-CGATGGAACTTCGACTTTGTCA-3' | 5'-GCACAAGGGTACAAGACAGTG-3' |
| PUMA | 5'-CGGAGACAAGAGGAGCAG-3' | 5'-GGAGTCCCATGATGAGATTG-3' |
| BTG2 | 5'-GCGTGAGCGAGCAGAGGCTT-3' | 5'-GGCTGGCCACCCTGCTGATG-3' |
| IL11 | 5'-ACTGCTGCTGCTGAAGACTC-3' | 5'-CCACCCCTGCTCCTGAAATA-3' |
| Ago2 | 5'-CGTGCCTGCTGGAATGTTTC-3' | 5'-CCATCCGTGAGGCCTGTATC-3' |

Supplementary Table 2. Antibodies in the study.

| Antibody | Company | Catalog # | Species | Dilution WB |
| --- | --- | --- | --- | --- |
| GAPDH | Proteintech | 60004-1-Ig | Mouse | 1:50000 |
| ENY2 | GeneTex | GTX629542 | Mouse | 1:1000 |
| β-tubulin | Cell signalling | 86298T | Mouse | 1:1000 |
| NPM1 | Proteintech | 10306-1-AP | Rabbit | 1:20000 |
| p53 | Proteintech | 10442-1-AP | Rabbit | 1:5000 |
| p21 | Proteintech | 10355-1-AP | Rabbit | 1:1000 |
| MDM2 | Proteintech | 66511-1-Ig | Mouse | 1:1000 |
| HA | Proteintech | 51064-2-AP | Rabbit | 1:5000 |
| STAT3 | Abcam | ab119352 | Mouse | 1:5000 |
| p-STAT3 | Abcam | ab76315 | Rabbit | 1:2000 |
| IL-11 | Abcam | ab187167 | Rabbit | 1:1000 |
| Ago2 | Proteintech | 67934-1-Ig | Mouse | 1:1000 |

Supplementary Table 3. siRNA sequences in the study.

| Name | Sequence (5’-3’) |
| --- | --- |
| siNC | UUCUCCGAACGUGUCACGUTT |
| siENY2-1 | GAAGGAGCTCCTACAAAGA |
| siENY2-2 | GATAGAAACTGGAGAAAGA |
| siENY2-3 | CTGGTACCTGACAGTGTAA |
| siNPM1-1 | GAAUUGCUUCCGGAUGACU |
| siNPM1-2 | GGAATGTTATGATAGGACA |
| siAgo2-1 | Forward: GCAAGAAGAGAUUAGCAAATT |
|  | Reverse: UUUGCUAAUCUCUUCUUGCTT |
| siAgo2-2 | Forward: GCUGAAUAUUGAUGUGUCATT |
|  | Reverse: UGACACAUCAAUAUUCAGCTT |
